# Innate and adaptive immunity associated with resolution of acute woodchuck hepatitis virus infection in adult woodchucks

**DOI:** 10.1371/journal.ppat.1008248

**Published:** 2019-12-23

**Authors:** Manasa Suresh, Stefanie Czerwinski, Marta G. Murreddu, Bhaskar V. Kallakury, Ashika Ramesh, Severin O. Gudima, Stephan Menne

**Affiliations:** 1 Department of Microbiology & Immunology, Georgetown University Medical Center, Washington, DC, United States of America; 2 Department of Pathology, Georgetown University Medical Center, Washington, DC, United States of America; 3 Department of Biochemistry and Molecular & Cellular Biology, Georgetown University Medical Center, Washington, DC, United States of America; 4 Department of Microbiology, Molecular Genetics & Immunology, University of Kansas Medical Center, Kansas City, KS, United States of America; Albany Medical College, UNITED STATES

## Abstract

Viral and/or host factors that are directly responsible for the acute *versus* chronic outcome of hepatitis B virus (HBV) infection have not been identified yet. Information on immune response during the early stages of HBV infection in humans is mainly derived from blood samples of patients with acute hepatitis B (AHB), which are usually obtained after the onset of clinical symptoms. Features of intrahepatic immune response in these patients are less studied due to the difficulty of obtaining multiple liver biopsies. Woodchuck hepatitis virus (WHV) infection in woodchucks is a model for HBV infection in humans. In the present study, five adult woodchucks were experimentally infected with WHV and then followed for 18 weeks. Blood and liver tissues were frequently collected for assaying markers of WHV replication and innate and adaptive immune responses. Liver tissues were further analyzed for pathological changes and stained for important immune cell subsets and cytokines. The increase and subsequent decline of viral replication markers in serum and liver, the elicitation of antibodies against viral proteins, and the induction of virus-specific T-cell responses indicated eventual resolution of acute WHV infection in all animals. Intrahepatic innate immune makers stayed unchanged immediately after the infection, but increased markedly during resolution, as determined by changes in transcript levels. The presence of interferon-gamma and expression of natural killer (NK) cell markers suggested that a non-cytolytic response mechanism is involved in the initial viral control in liver. This was followed by the expression of T-cell markers and cytolytic effector molecules, indicating the induction of a cytolytic response mechanism. Parallel increases in regulatory T-cell markers suggested that this cell subset participates in the overall immune cell infiltration in liver and/or has a role in regulating AHB induced by the cytolytic response mechanism. Since the transcript levels of immune cell markers in blood, when detectable, were lower than in liver, and the kinetics, except for NK-cells and interferon-gamma, did not correlate well with their intrahepatic expression, this further indicated enrichment of immune cells within liver. Conclusion: The coordinated interplay of innate and adaptive immunity mediates viral clearance in the woodchuck animal model of HBV infection. The initial presence of NK-cell associated interferon-gamma response points to an important role of this cytokine in HBV resolution.

## Author summary

Hepatitis B virus (HBV) is a major public health concern and 257 million chronic carriers worldwide are at risk of developing serious liver disorders. Current treatment options for chronic hepatitis B (CHB) are rarely inducing viral clearance. HBV infection in immunocompetent adults is mostly asymptomatic, with a less than 5% chance of progressing to chronic infection. Contrary, more than 95% of infants infected with HBV at birth or during early childhood develop chronic infection. The viral and host factors directly deciding over the acute *versus* chronic outcome are largely unknown. Understanding the features of innate and adaptive immune responses during acute, self-limiting HBV infection will aid the understanding of underlying mechanisms responsible for HBV resolution, and thus support the design of immunomodulatory drugs for treatment of CHB. However, studying acute hepatitis B (AHB) in humans is challenging due to its asymptomatic nature and the limitations in obtaining multiple liver biopsies. Infection of adult woodchucks with the HBV-like woodchuck hepatitis virus (WHV) is a suitable model for studying AHB. Our comprehensive work describes the kinetics of viral replication and associated innate and adaptive immune responses, both in liver and blood, during resolution of AHB in the woodchuck model.

## Introduction

The annual mortality caused by viral hepatitis infections is on the rise globally, with a major contribution by hepatitis B virus (HBV) [1]. HBV infection can lead to chronic hepatitis B (CHB), with severe liver disorders, including cirrhosis and hepatocellular carcinoma (HCC). More than 95% of HBV infections in adults are self-limited where individuals usually develop acute hepatitis B (AHB) and resolve thereafter, with long-lasting control by humoral and cellular immune responses [2]. In contrast, the same percentage of newborns and infants infected with HBV develops CHB [3]. Although the mature and robust immune system of adults is thought to be mainly responsible for the high rate of resolution, specific viral and/or host factors and mechanisms deciding over the acute *versus* chronic outcome of HBV infection in newborns are investigated [4,5], but remain largely unknown. The course of acute HBV infection is characterized by an initial peak in viral DNA and proteins, such as HBV surface (HBsAg) and e antigens (HBeAg). This is followed by a rise in liver enzymes, with mainly alanine aminotransferase (ALT) assayed, but also others, including sorbitol dehydrogenase (SDH) and aspartate aminotransferase (AST). Antibodies to HBV core antigen (anti-HBc antibodies) can be detected before the onset of clinical symptoms. The reduction in HBV DNA and the loss of HBsAg achieved during resolution is accompanied by the generation of antibodies to HBsAg (anti-HBs antibodies) [3]. HBeAg is also cleared by antibodies to HBeAg (anti-HBe antibodies). However, in individuals who acquire HBV infection *via* vertical transmission or during early childhood, and who predominantly develop chronic infection later in life, HBV DNA and antigens continue to persist at high levels, without detectable anti-HBs antibodies, eventually leading to various stages of chronicity and liver diseases.

Current treatment regimens for CHB include prolonged administration of nucleos(t)ide analogs (mainly entecavir and tenofovir) or of an immunomodulator (i.e., pegylated interferon-alpha (IFN-α)). However, treatment with these antivirals leads to functional cure or even viral clearance in only a small subset of patients, and therapy, especially with IFN-α, is sometimes associated with severe side effects [6]. For overcoming these limitations, new drug targets and treatment strategies are currently developed. Understanding the complex immune response mediating natural resolution of HBV infection is an important approach to characterize in more detail the immunodeficiencies present in chronic conditions, and to understand what future treatments based on immunomodulation need to achieve for bringing out an antiviral effect. The analysis of blood samples from patients who developed AHB has provided insight into many aspects of the immune response, including the role of natural killer (NK) cells during the early stages of infection, the importance of cytokines such as interleukin (IL) 12, IL-18, tumor necrosis factor-alpha (TNF-α), and interferon-gamma (IFN-γ) during acute infection, and the broad and multi-specific responses of helper T-cells (Th-cells) and cytolytic T-cells (CTLs) during resolution [7–10]. Studying AHB in humans, however, is challenging since the early weeks after HBV infection are mostly asymptomatic, and hence there is limited data available on the immune response during the early preclinical phase of infection. Furthermore, current knowledge on AHB in humans is mainly derived from blood samples, and the kinetics of innate and adaptive immune responses in liver are less characterized due to the difficulty of obtaining multiple liver biopsies from the same individual.

The Eastern woodchuck (*Marmota monax*) is naturally infected with the woodchuck hepatitis virus (WHV), a mammalian hepadnavirus, which is closely related to human HBV [11]. Neonatal WHV infection parallels the main route of human (vertical) transmission of chronic HBV infection and displays a disease course comparable to that observed in HBV-infected patients. Adult WHV infection almost always leads to an acute, self-limited outcome, similar to HBV infection in adult humans. Thus, acute and chronic WHV infections in woodchucks represent a fully immunocompetent model for studying innate and adaptive immune responses associated with resolution *versus* progression to chronicity, including CHB and HCC [11].

Previous studies in woodchucks have investigated selected aspects of the immune response and showed that resolution of WHV infection is associated with strong T-cell proliferation and IFN-γ and TNF-α expression during the early phase of infection, and with seroconversion to virus-neutralizing antibodies targeting WHV surface antigen (WHsAg), while progression to chronicity involves deficiencies in these T- and B-cell responses [12–14]. The investigation of intrahepatic immune markers, however, has provided sometimes contrasting results regarding the activation of innate immune response by WHV during the early stages of infection [15,16]. Thus, a single study with comprehensive analysis and comparison of innate and adaptive immunity in both the periphery and liver during resolution of WHV infection is wanted. Here, the kinetics of WHV replication and associated responses of WHV-specific T- and B-cells and the expression of immune response markers were investigated in five adult woodchucks. The overall results revealed that the coordinated interplay of innate and adaptive immunity mainly in liver drives resolution of WHV infection in woodchucks, and further indicated an important role of NK-cell associated IFN-γ response in the initial viral control following infection.

## Results

### Course of acute, self-limited WHV infection in the periphery and liver of woodchucks

**Fig 1:**
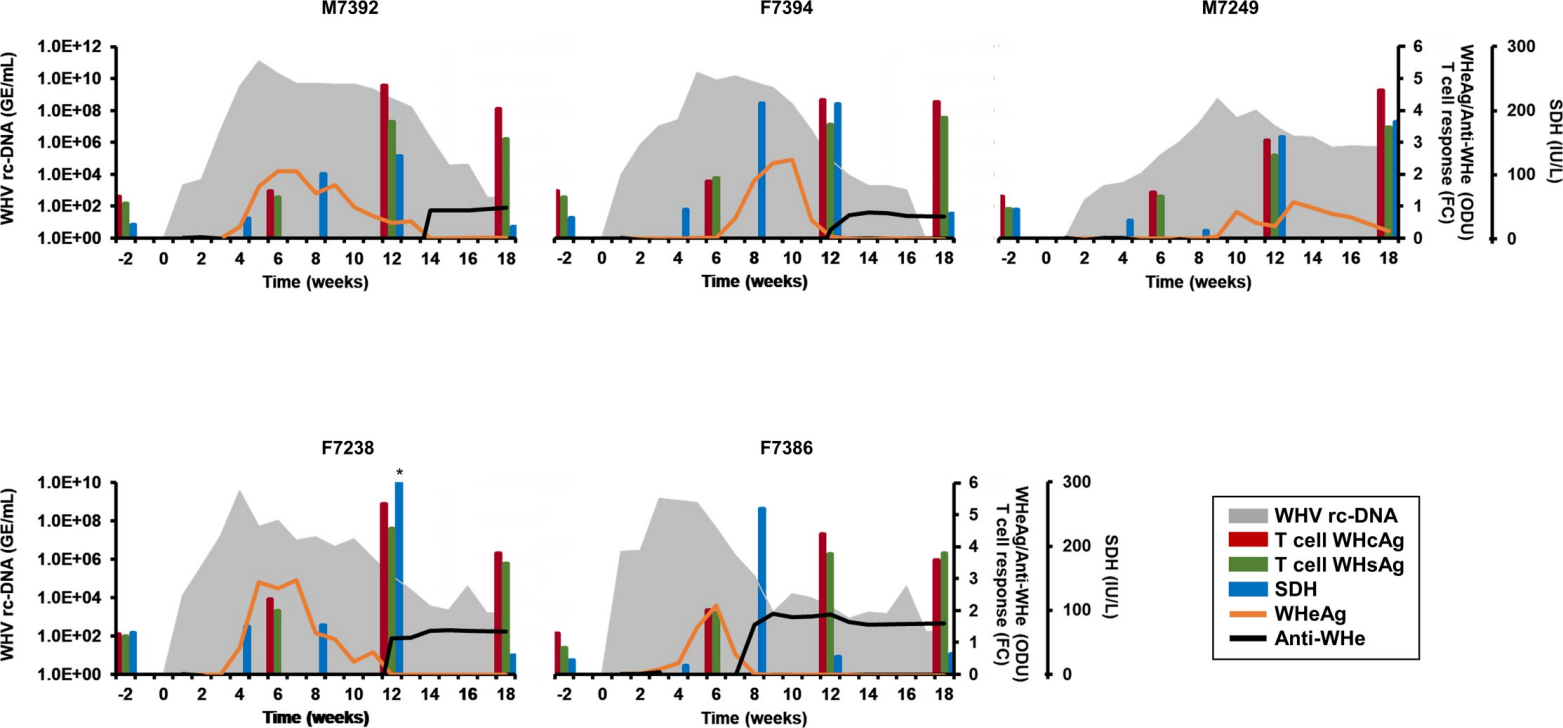
Course of resolving WHV infection in the periphery. Kinetics of serum viremia (WHV rc-DNA), antigenemia (WHeAg), antibody response (anti-WHe antibodies), activity of liver enzymes (SDH), and T-cell responses (specific to WHcAg and WHsAg). Changes in WHV rc-DNA are plotted on the left y-axis, while changes in SDH, WHeAg, anti-WHe antibodies, and WHcAg- and WHsAg-specific T-cell responses are plotted on the right y-axes. *, serum SDH activity was 432 IU/L; GE, genomic equivalents; IU, international unit, ODU, optical density unit, FC, fold-change in T-cell proliferation from unstimulated controls.

The five woodchucks of the present study were a subgroup of animals that were experimentally infected with an equal dose of serum-derived WHV [17]. These animals were selected based on the duration of infection, and thus allowed to dissect in more detail the kinetics of innate and adaptive immune responses. Following the inoculation, the course of acute, self-limited (i.e., resolving) WHV infection in these animals was determined in blood and liver as largely described [17]. In the periphery, as already reported [17] and represented in Fig 1, serum WHV relaxed-circular DNA (rc-DNA) was detectable in all woodchucks as early as week 1 or 2, and peak viremia was observed during weeks 3–5, except for M7249, in which viremia was maximal at week 9. Thereafter, viremia declined, but did not become undetectable in most woodchucks by the end of the study at week 18, except for F7394 with absent WHV rc-DNA at this time. Serum WHsAg was detectable less frequently than WHV rc-DNA, but peak antigenemia was noted during weeks 2–9 in all animals around the time of maximum viremia [17]. Serum antibodies to WHsAg (anti-WHs antibodies) were elicited in all woodchucks starting as early as week 3, and peak antibody response was observed during weeks 13–16 [17]. The mounting antibody response, as reflected by increasing titers, coincided with the initial and then marked decline in viremia in most woodchucks. The peak in serum WHV e antigen (WHeAg) was observed during weeks 6–13 (Fig 1), and thus later than the maximum in WHsAg in all animals [17]. Following the decline in antigenemia, antibodies to WHeAg (anti-WHe antibodies) were noted in most animals, starting as early as weeks 8–14, with a peak during weeks 12–15 (Fig 1). T-cell responses to WHsAg and WHV core antigen (WHcAg) were developed in all woodchucks (Fig 1). WHcAg-specific T cell response was considered present in F7238 at week 6, as the change from baseline was above 2.1. All other woodchucks presented with WHV-specific T cell responses starting at week 12. Peak T-cell responses were observed at week 12 in M7392 and F7238, at weeks 12 and 18 in F7394 and F7386, and at week 18 in M7249 (fold-change: 2.3–5.3), and correlated with the marked decline in viremia in most animals.

In the liver, as already reported [17], WHV replicative intermediate DNA (RI-DNA), covalently-closed circular DNA (cccDNA) and pre-genomic RNA (pgRNA) were detectable in all woodchucks at week 5. Peak WHV replication was observed during weeks 5–13. Thereafter, viral DNA and RNA molecules declined in liver, but did not become undetectable by the end of the study. Correlating with the hepatic WHV replication and expression of WHcAg [17], hepatocytes positive for the cytoplasmic (cyt) location of WHsAg were present in all woodchucks at week 5 (Table 1 and S1 Fig). Peak WHsAg expression was observed during weeks 9–13 (range: 5.6–18.3% of hepatocytes positive for cytWHs). Thereafter, WHsAg expression declined and became absent during weeks 13–18, except for F7249, in which about 3% of hepatocytes were still positive for this antigen at the end of the study. Correlating further with the peripheral decline in viremia (Fig 1), but temporally more associated with the initial and then marked reduction of hepatic WHV DNA and RNA [17], serum activity of SDH increased starting at weeks 8–12 and/or was maximal elevated during weeks 8–18 (range: 128–432 international units (IU)/L) (Fig 1 and Table 1). Serum gamma-glutamyl transferase (GGT) activity was unchanged in all animals, except for F7238 with a transient increase at week 4 (18 IU/L). Peak serum activity of liver enzymes was temporally associated with moderate to marked sinusoidal and portal hepatitis/inflammation in liver, all of which is indicative of AHB in woodchucks (Table 1). Liver cell proliferation as determined by staining for the cellular proliferation marker Ki67 peaked during this time as well (range: 1.9–6.7% of hepatocytes positive) (Table 1 and S2 Fig), indicating increased replenishment of liver with hepatocytes. Although individual variability in the kinetics (and levels) was noted for the above viral and host markers, especially for M7249, which experienced a longer-lasting viremic phase than all other animals, the overall results suggested that the five outbred woodchucks followed in this study developed an acute, self-limited WHV infection, which eventually resolved.

**Table 1:** Kinetics of hepatic WHsAg expression, liver hepatitis/inflammation, and hepatocyte proliferation in woodchucks.

		Marker change in liver at the indicated weeks during the course of WHV resolution				
Woodchuck	Marker	-2	5	9	13	18
M7392	Cyt WHsAg^a^	0	0.6	8.1	7.1	0
F7394		0	1.3	13.7	3.6	0
M7249		0	0.8	1.1	16.7	3.3
F7238		0	1.2	12.3	18.3	0
F7386		0	1.8	5.6	1.3	0
M7392	Hepatitis^b^	Mild	Absent	Moderate	Moderate*	Moderate
F7394		Absent	Absent	Marked*	Moderate	Absent
M7249		Absent	Absent	Absent	Moderate	Moderate*
F7238		Absent	Absent	Moderate	Moderate*	Mild
F7386		Absent	Mild	Moderate*	Moderate	Absent
M7392	Ki67^c^	0.2	0.4	3.1	2.6	0.4
F7394		0.3	0.3	1.9	0.7	0.1
M7249		0.3	0.2	0.8	2.6	6.0
F7238		0.2	0.6	6.7	5.2	1.0
F7386		0.3	0.9	2.3	1.0	0.3

^a^ Percentage of hepatocytes positive for cytoplasmic (cyt) WHsAg.

^b^ Composite score for sinusoidal and portal hepatitis/inflammation. Peak serum SDH activity is indicated by an asterisk.

^c^ Percentage of hepatocytes positive for the cellular proliferation marker Ki67.

### Kinetics of immune markers in liver and periphery associated with resolution of WHV infection in woodchucks

#### Type Ⅰ interferons (IFNs) and interferon-stimulated genes (ISGs)

**Fig 2:**
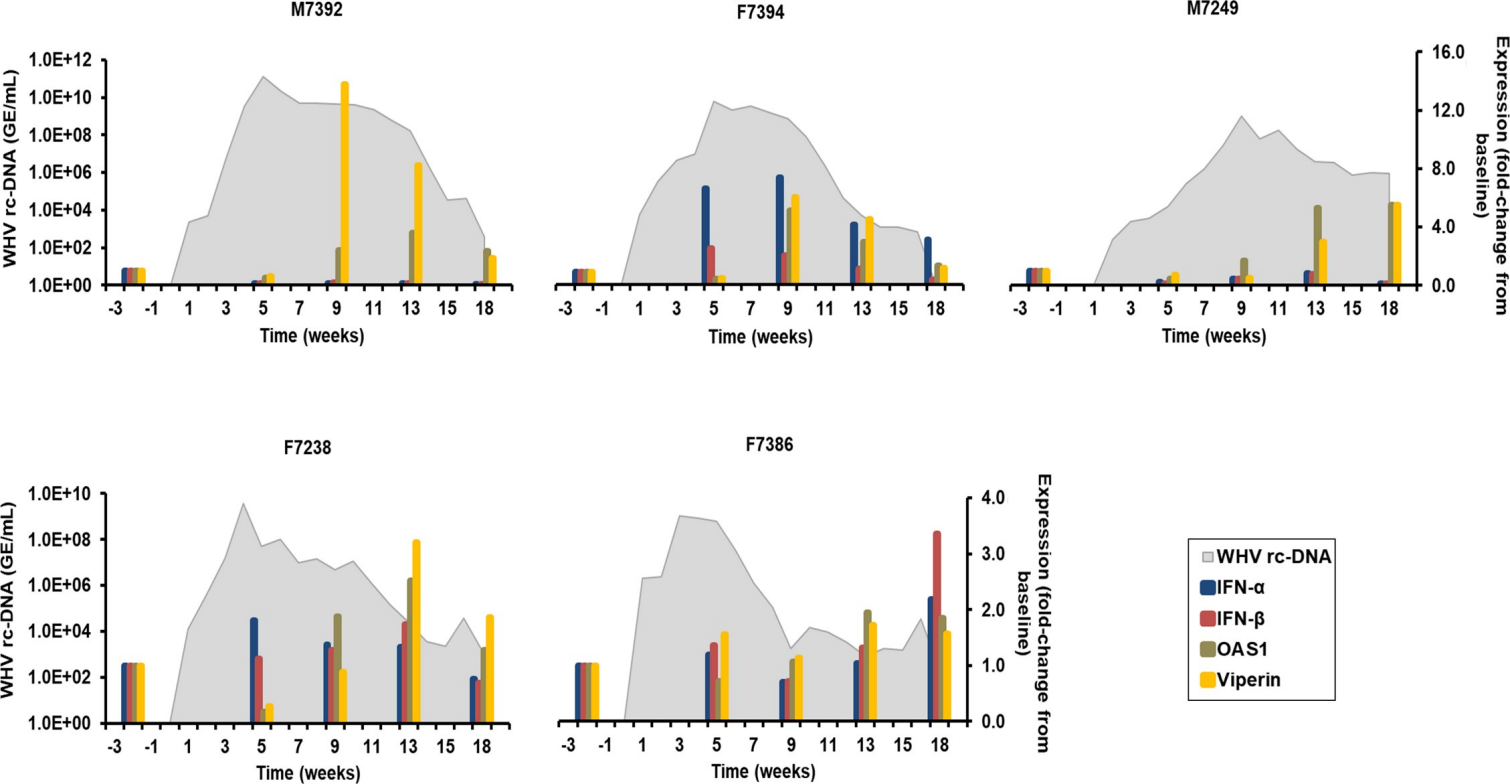
Intrahepatic expression of type I IFNs and ISGs. Changes in the expression of IFN-α, IFN-β, OAS1, and viperin in the liver. The fold-change in transcript level of genes from baseline is plotted on the right y-axis, while serum WHV rc-DNA loads are plotted on the left y-axis.

Type Ⅰ IFNs secreted by immune and non-immune cells play an important role in the host defense against viral infection *via* subsequent induction of ISGs [18]. For characterizing the innate immune response in woodchucks induced by WHV, the expression of type Ⅰ IFNs (IFN-α/β) and two ISGs (viperin (RSAD2) and 2’-5’-oligoadenylate synthetase 1 (OAS1)) in liver and periphery was determined. In the liver (Fig 2), peak expression of IFN-α and IFN-β was observed during weeks 5–18 in F7394 and F7386 (fold-change: IFN-α, 2.2–8.1; IFN-β, 2.8–3.4). Although sometimes detectable, type Ⅰ IFN expression in the other animals was considered absent, as the change from baseline was below 2.1. Nevertheless, OAS1 expression was induced in these animals and peaked during weeks 9–18 in M7392, F7394, M7249, and F7238 (fold-change: 2.5–5.6). Peak expression of viperin was noted during weeks 9–18 in the same animals (fold-change: 3.2–14.3). ISG expression in F7386 was detectable as well, especially at weeks 13–18, but considered absent, because the fold-change was below 2.1. Since the induction of type I IFNs and subsequently of ISGs is typically transient, and thus difficult to measure, it is likely that their peak expression was missed in some of the woodchucks due to the timing of liver tissue collection. In the periphery (S3 Fig), the peak expression of type I IFNs was observed during weeks 8–16 in M7392, F7394, M7249, and F7238, with IFN-β predominantly induced (fold-change: IFN-α, 6.5–13.4; IFN-β, 6.5–52.2), but not in F7386, despite of IFN-α/β expression in liver. Viperin expression was maximal during weeks 4–12 in M7392, F7394, M7249, and F7238 (fold-change: 4.4–8.2). OAS1 expression was considered detectable only in F7238 at week 4 (fold-change: 2.6). Based on the presence of transcripts for type I IFNs and two ISGs that was usually weaker in blood than in liver, when detectable, and the maximum expression of these and most other immune response markers tested, a correlation was not apparent. It further appeared that the investigated innate immune response markers were not markedly induced by WHV immediately after the inoculation in both compartments of most woodchucks.

#### NK-cell markers

**Fig 3:**
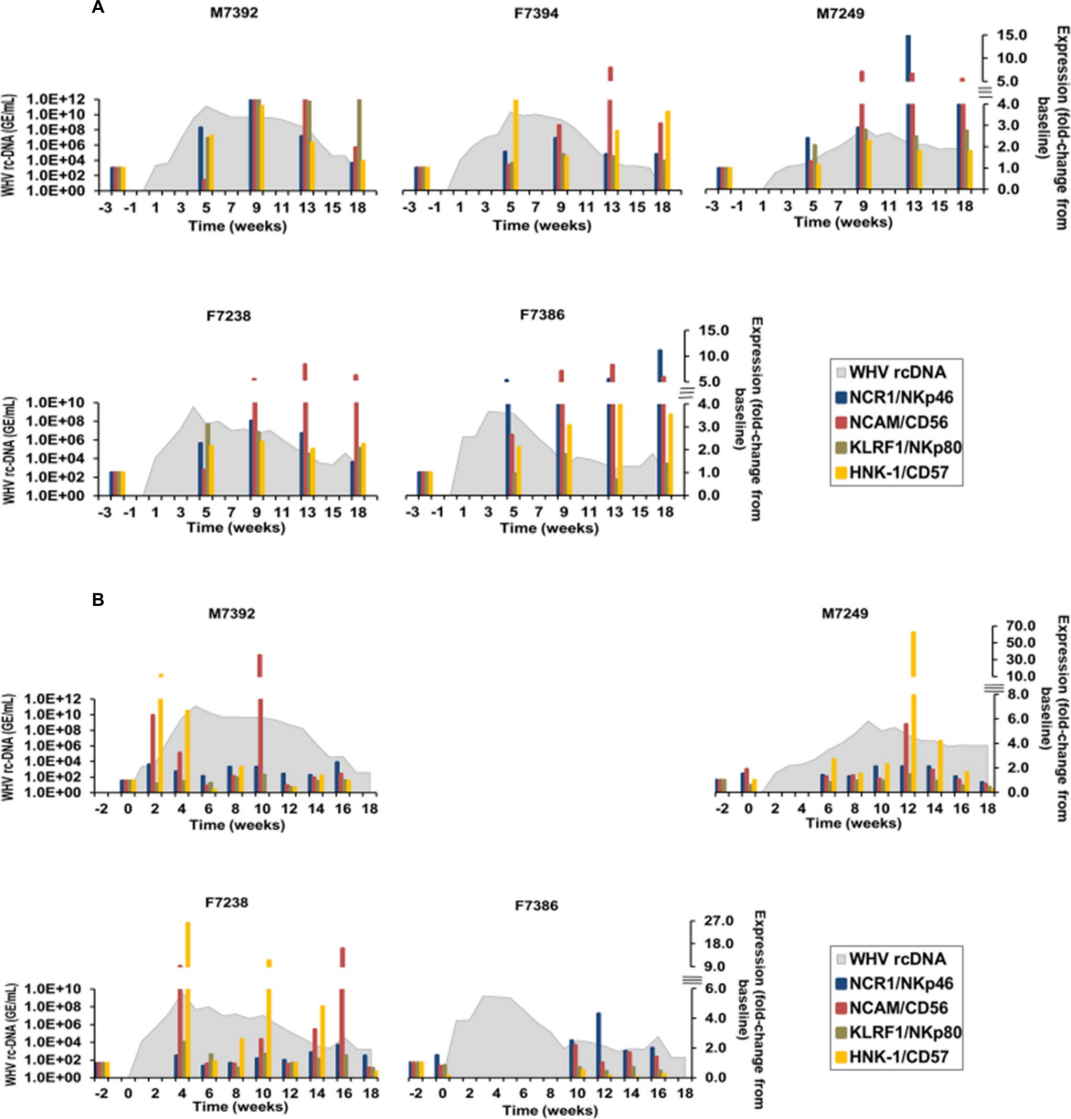
Intrahepatic and peripheral expression of NK-cell markers. (**A**) Changes in the expression of NCR1/NKp46, NCAM/CD56, KLRF1/NKp80, and HNK-1/CD57 in the liver. (**B**) Changes in the expression of NCR1/NKp46, NCAM/CD56, KLRF1/NKp80, and HNK-1/CD57 in the periphery. In (A) and (B), the fold-change in transcript level of genes from baseline is plotted on the right y-axis, while serum WHV rc-DNA loads are plotted on the left y-axis.

For further characterizing the immune response in woodchucks after WHV inoculation, NK-cells were included, since this cell subset presents a major component of the innate immunity, which plays an important role in IFN-γ production along with immune surveillance by identifying and killing virus-infected cells [19,20]. Thus, the expression of selected NK-cell markers, including natural cytotoxicity triggering receptor 1 (NCR1 or NKp46), neural cell adhesion molecule (NCAM or CD56), killer cell lectin-like receptor F1 (KLRF1 or NKp80), and human natural killer-1 receptor (HNK-1 or CD57) was assayed in blood and liver (Fig 3). In the liver, peak expression of NCR1/NKp46 was noted during weeks 9–13 in all animals (fold-change: 2.3–14.9), except for F7386 at week 18 (fold-change: 11.1). NCAM/CD56 expression peaked at week 9 in M7249 (fold-change: 7.1) and at week 13 in M7392, F7394, F7238, and F7386 (fold-change: 4.8–8.3). KLRF1/NKp80, another NK-cell activation marker, showed peak expression during weeks 9–18 in M7392 and M7249 (fold-change: 2.8–4.9) and at week 5 in F7238 (fold-change: 3.1). In F7394 and F7386, expression of this receptor remained close to baseline. HNK-1/CD57 expression was maximal at week 5 in F7394 (fold-change: 4.7), at week 9 in M7392, M7249, and F7238 (fold-change: 2.3–3.7), and at week 13 in F7386 (fold-change: 4.4). Of note is that expression increases of the NK-cell activation receptors NCR1/NKp46 and KLRF1/NKp80 and the NK-cell surface receptor HNK-1/CD57 were already observed at week 5 in M7392, M7249, F7238, and F7386 (fold-change: 2.3–5.3), in M7249, F7238, and F7386 (fold-change: 2.1–3.1), or in M7392, F7394, F7238, and F7386 (fold-change: 2.1–4.7), respectively. In addition to these cell surface markers, the expression of activation receptors (killer cell lectin-like receptor K1 (KLRK1 or NKG2D) and cluster of differentiation (CD) 16 (CD16)) and one inhibitory receptor (killer cell lectin-like receptor C1 (KLRC1 or NKG2A)) (S4 Fig) were measured that are also well-described markers for NK-cells [21]. All additionally tested NK-cell markers showed peak expression in liver during the marked decline of WHV replication. In blood, testing of all NK-cell markers was not possible for all timepoints due to insufficient RNA amounts for some of the animals; however, nearly complete data sets are presented for four woodchucks (M7392, M7249, F7238, and F7386). The expression of NCR1/NKp46 and NCAM/CD56 was maximal during weeks 10–16 in these four animals (fold change: NCR1/NKp46, 2.1–4.3; NCAM/CD56, 2.2–36.9). KLRF1/NKp80 expression stayed close to baseline in all animals, except for F7238 at week 4 (fold-change: 2.6). HNK-1/CD57 expression peaked during weeks 2–12 in M7392, M7249, and F7238 (fold-change: 10.2–62.0). Similar to liver, the additional NK-cell markers measured in blood (S4 Fig) showed peak expression during the marked decline of serum viremia. Although the magnitude of (peak) expression differed between liver and blood, the kinetics of NK-cell markers obtained in both compartments were overall comparable for most woodchucks.

#### Antigen presenting cell (APC) markers

**Fig 4:**
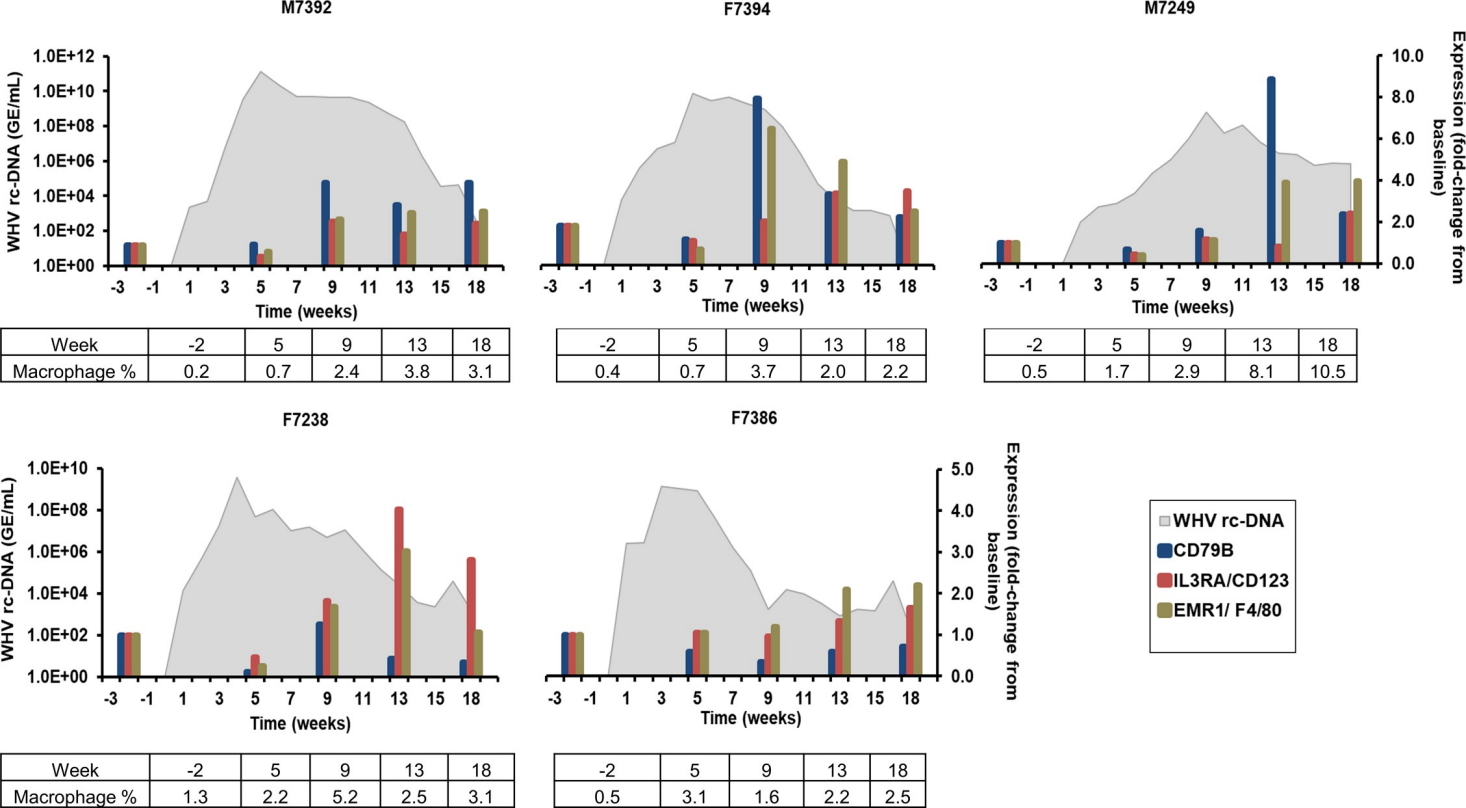
Intrahepatic expression of APC markers. Changes in the expression of CD79B (B-cell), IL3RA/CD123 (pDC), and EMR1/F4/80 (macrophage) in the liver. The fold-change in transcript level of genes from baseline is plotted on the right y-axis, while serum WHV rc-DNA loads are plotted on the left y-axis. Changes in the percentage of macrophages in liver are provided at the bottom of each graph.

APCs such as dendritic cells (DCs), macrophages, and B-cells were further studied, since these cell subsets are responsible for initiating and shaping the adaptive immune response [22]. The kinetics of these immune cells were determined by the intrahepatic and peripheral expression of selected markers, including CD79B for B-cells, interleukin 3 receptor A (IL3RA or CD123) for plasmacytoid DCs (pDCs), and EGF-like module-containing mucin-like hormone receptor like-1 (EMR1 or F4/80) for macrophages. In the liver (Fig 4), CD79B expression peaked during weeks 9–13 in M7392, F7394, and M7249 (fold-change: 4.0–8.9), although the first animal also had a pronounced expression at week 18. In the other two woodchucks, CD79B expression was nearly unchanged. Peak IL3RA/CD123 expression was observed during weeks 9–18 in M7392, M7249, and F7238 (fold-change: 2.1–4.0). The other two animals had absent marker expression. EMR1/F4/80 expression was maximal during weeks 9–18 in all woodchucks (fold-change: 2.2–3.9). The changes in the percentage of macrophages, as determined by immunostaining, correlated well with the kinetics of EMR1/F4/80 expression in most woodchucks, except for F7238, in which peak expression was noted at week 13, while the highest percentage of macrophages was obtained at week 9 (Fig 4 and S5 Fig). Contrary to the findings in liver, APC marker expression in blood was rarely positive (S6 Fig). Exceptions included the expression of CD79B at week 12 in M7249 (fold-change: 2.1), IL3RA/CD123 at week 4 in F7238 (fold-change: 2.6), and EMR1/F4/80 at week 10 in M7392 (fold-change: 2.6). In F7386, based on the timepoints analyzed, expression of IL3RA/CD123 and CD79B appeared maximal at weeks 4 or 10, respectively (fold-change: 2.5 and 2.4). Data on peripheral APC markers were not available for F7394. Overall, a correlation of APC marker expression in liver and blood was not apparent, and especially not for peripheral CD79B expression and the elicitation of anti-WHs and anti-WHe antibodies.

#### T-cell markers

**Fig 5:**
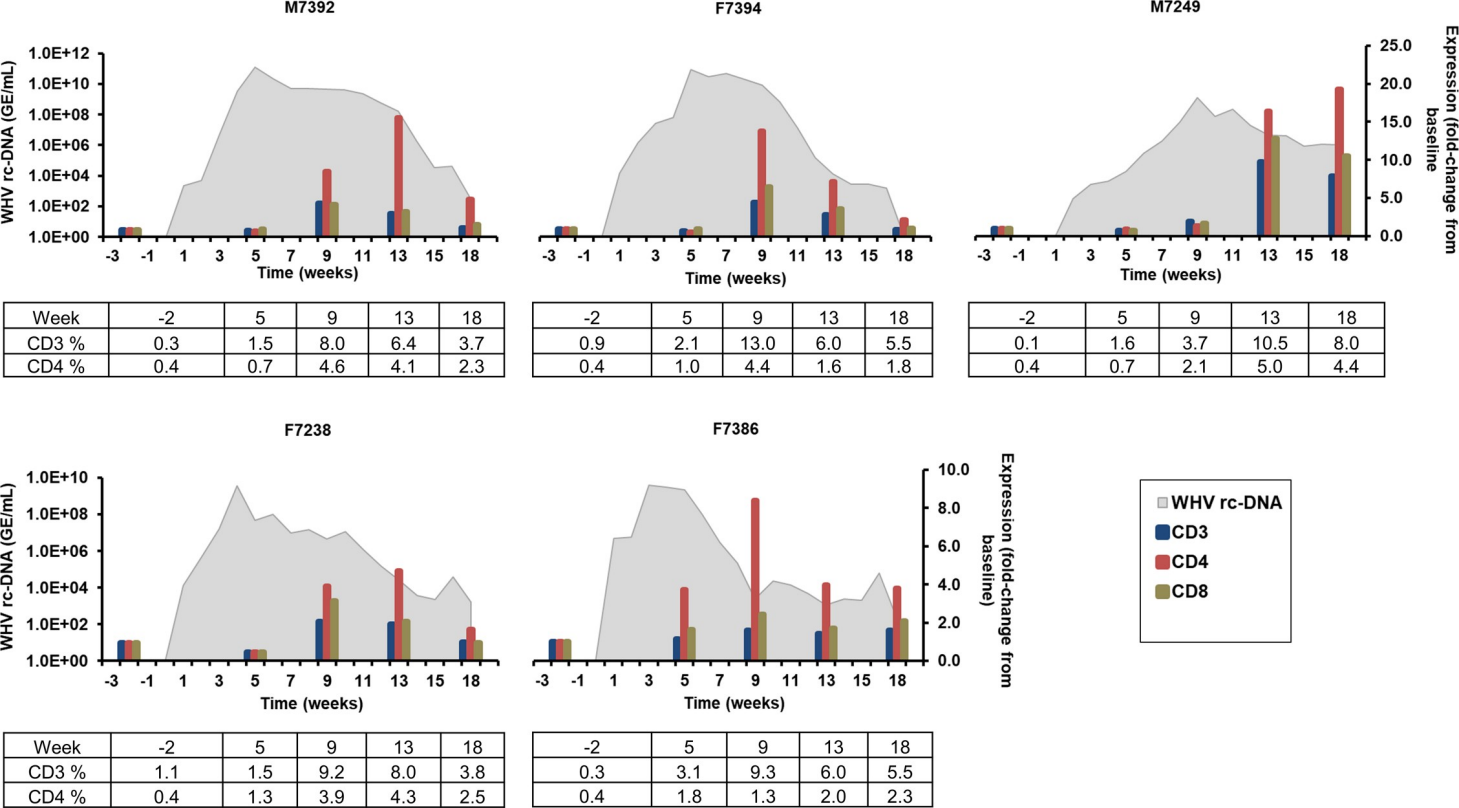
Intrahepatic expression of T-cell markers. Changes in the expression of CD3, CD4, and CD8 in the liver. The fold-change in transcript level of genes from baseline is plotted on the right y-axis, while serum WHV rc-DNA loads are plotted on the left y-axis. Changes in the percentage of CD3- and CD4-positive cells in liver are provided at the bottom of each graph.

The induction of adaptive immunity, including the activation of T-cells, is critical for mounting a strong antiviral immune response against HBV [23]. CD4+ Th-cells and CD8+ CTLs are the key effector cells involved in recognizing and killing of virus-infected hepatocytes. Furthermore, Th-cells produce a wide range of cytokines upon activation, which are responsible for keeping the antiviral immune response active, and which influence the maturation of B-cells [23]. In the liver (Fig 5), the peak in CD3 expression was observed during weeks 9–13 in M7392, F7394, M7249, and F7238 (fold-change: 2.2–9.8). CD3 expression stayed close to baseline in F7386. Maximum CD4 expression was noted during weeks 9–18 in all woodchucks (fold-change: 4.9–19.3), while CD8 expression peaked during weeks 9–13 in these animals (fold-change: 2.4–12.9). The changes in the percentage of CD3- and CD4-positive cells, as determined by immunostaining, correlated well with the transcription levels of both T-cell markers in liver, and more so for CD3 than for CD4 (Fig 5, S7 Fig, and S8 Fig). CD8-positive cells could not be stained, because a cross-reactive antibody has not been identified yet. Intrahepatic peak expression of the above T-cell markers coincided with the initial, and more so with the marked decline of WHV replication [17]. Due to the applied assay and the lack of an anti-CD8 antibody, T-cells specific for WHcAg and WHsAg could not be further differentiated into CD4+ and CD8+ T-cells. In the periphery (S9 Fig), T-cell marker expression was nearly unchanged, except for peak CD3 expression at weeks 2 and 18 in F7394 or F7386, respectively (fold-change: 2.4–2.5), for peak CD4 expression at weeks 2 and 14 in F7238 or F7394, respectively (fold-change: 4.3–2.2), and for peak CD8 expression at week 16 in F7394 and F7238 (fold-change: 2.3–2.9).

#### CTL markers

**Fig 6:**
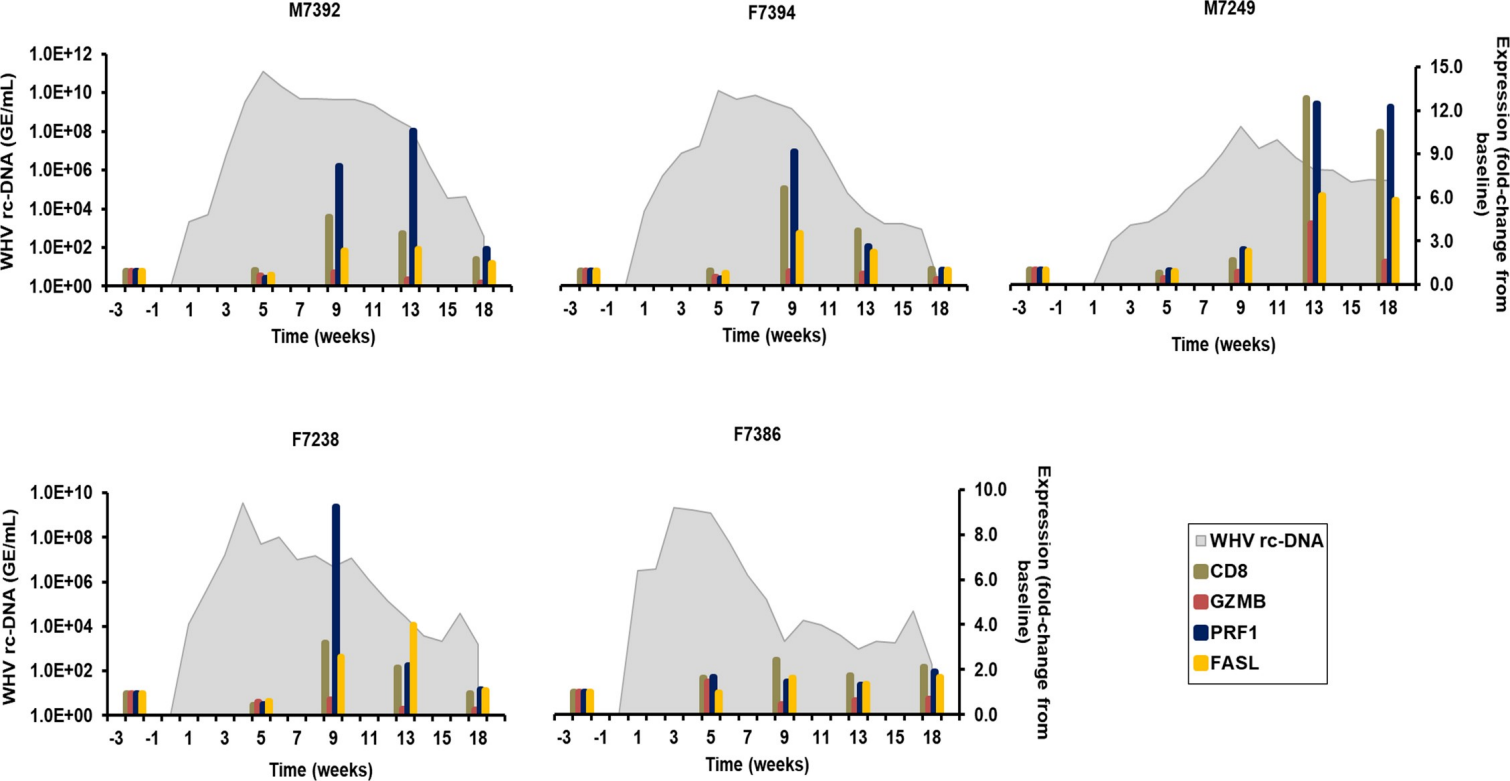
Intrahepatic expression of markers for CD8+ T-cells and cytolytic effector molecules. Changes in the expression of CD8, GZMB, PRF1, and FASL in the liver. The fold-change in transcript level of genes from baseline is plotted on the right y-axis, while serum WHV rc-DNA loads are plotted on the left y-axis.

For investigating in more detail the role of CD8+ T-cells in WHV resolution, the effector function of mainly CTLs (but also of NK-cells) was analyzed, because these cells can release cytotoxins, such as granzyme B (GZMB) and perforin (PRF1), or interact with the Fas receptor on virus-infected hepatocytes with their Fas ligand (FASL) [24]. Thus, the expression of cytolytic effector molecules was determined and correlated with the CD8 transcript levels described above. In liver (Fig 6), GZMB expression stayed close to baseline in all woodchucks, except for M7249 with a peak at week 13 (fold-change: 4.2). Expression of PRF1 and FASL was more frequently detected, and was maximal during weeks 9–13 in M7392, F7394, F7249, and F7238 (fold-change: PRF1, 8.9–12.4; FASL, 2.4–6.1). Peak expression of both markers correlated well with that of CD8, and again coincided with the decline in WHV replication [17]. In the periphery (S10 Fig), the expression of GZMB in blood was maximal during weeks 10–16 in F7394, M7249, F7238, and F7386 (fold-change: 2.2–5.4). During the same time, peak expression of PRF1 was observed in M7392, M7249, F7238, and F7386 (fold-change: 2.3–4.6). PRF1 expression in F7394 was not determined due to insufficient RNA amount. Contrary to liver, FASL expression was nearly unchanged in all animals, except for F7386 with peak expression at week 16 (fold-change: 2.4-fold).

#### Regulatory T-cell (T_reg_) markers

**Fig 7:**
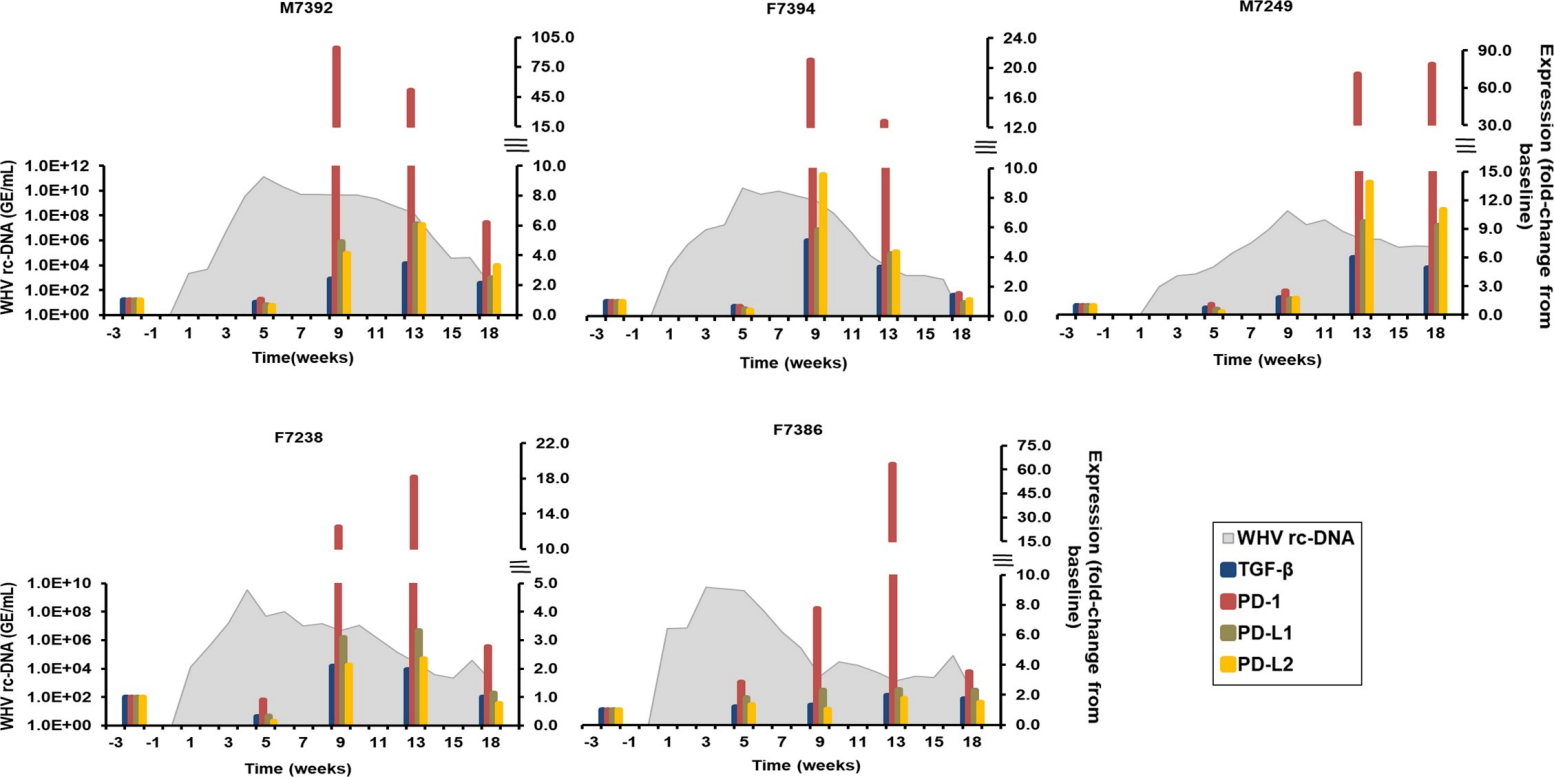
Intrahepatic expression of T_reg_ markers. Changes in the expression of TGF-β, PD-1, PD-L1, and PD-L2 in the liver. The fold-change in transcript level of genes from baseline is plotted on the right y-axis, while serum WHV rc-DNA loads are plotted on the left y-axis.

For counteracting and balancing the effector function of T-cells against viruses, the immune system has developed specific regulatory mechanisms. Molecules involved in regulating the T-cell response include the transforming growth factor-beta (TGF-β) that controls cell growth, differentiation and proliferation, the programmed cell death-1 receptor (PD-1) that is expressed on activated effector T-cells, and the programmed cell death-1 ligands 1 and 2 (PD-L1 and PD-L2) that are expressed on APCs [10,25]. In liver (Fig 7), peak TGF-β expression was observed during weeks 9–13 in all woodchucks (fold-change: 2.1–6.0), except for F7386. The inhibitory receptor, PD-1, showed maximal expression during weeks 9–18 in all animals (fold-change: 18.2–94.5). Furthermore, peak expression of the ligands, PD-L1 and/or PD-L2, was noted during weeks 9–13 in almost all woodchucks (fold-change: PD-L1, 2.4–9.7; PD-L2, 2.4–13.9). The only exception was F7386, with a PD-L2 expression close to baseline. Overall, maximum T_reg_ marker transcript levels correlated with the maximum expression of cytolytic effector molecules, elevated liver enzyme activity in serum, and pronounced hepatis/inflammation in liver, indicating that T_regs_ are a part of the overall immune cell infiltration in liver and/or have a role in regulating the liver injury induced by the adaptive immune response. In the periphery (S11 Fig), the expression of T_reg_ markers was rarely positive, except for PD-1 at week 8 in F7394 (fold-change: 2.7) and at week 12 in M7249 and F7238 (fold-change: 2.1–2.9) and for PD-L2 at week 14 in M7249 (fold-change: 2.3).

#### Comparison of NK- and T-cell markers in liver

**Fig 8:**
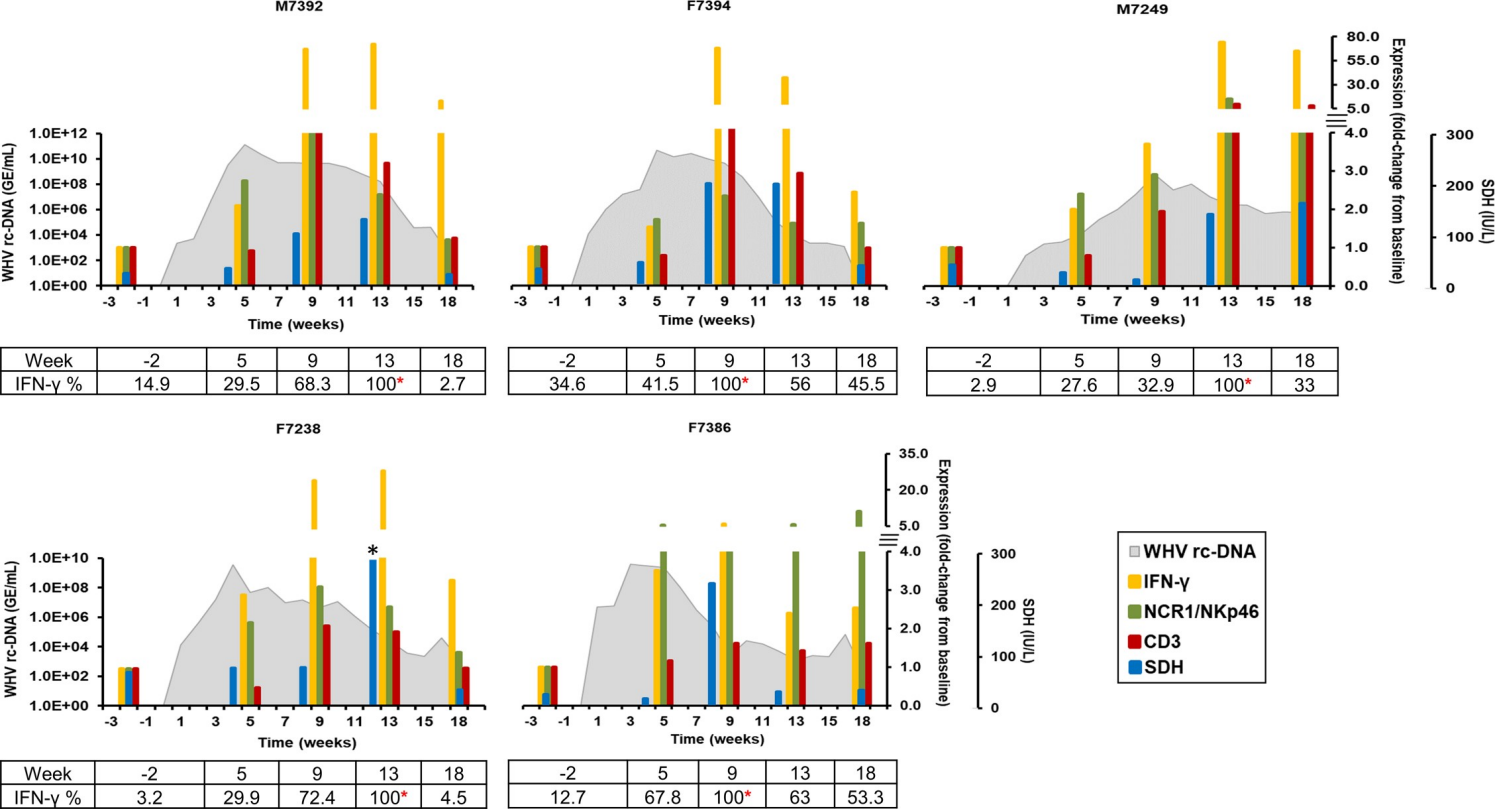
Comparison of intrahepatic expression of NK- and T-cell markers and IFN-γ with serum SDH activity. The fold-change in liver transcript level of NCR1/NKp46, CD3, and IFN-γ from baseline and the change in serum levels of SDH are plotted on the right y-axes, while serum WHV rc-DNA loads are plotted on the left y-axis. By setting the maximum average mean intensity at 100% (also indicated by an asterisk), the relative percentages of IFN-γ staining intensity in liver are provided at the bottom of each graph. *, serum SDH activity was 432 IU/L. IU, international unit.

For further delineating the sequence of immune response events involved in WHV resolution, the induction of NK- and T-cells within liver was compared and correlated with the intrahepatic expression of IFN-γ and the serum activity of SDH (Fig 8). Expression of NCR1/NKp46, an activation receptor commonly used for identifying NK-cells, increased at week 5 in M7392, M7249, F7238, and F7386. At this time, CD3, a marker for identifying T-cells in general, was unchanged in all woodchucks and expression stayed close to baseline. Serum SDH levels fluctuated somewhat, but were not elevated in any animal at this time. The expression increases in NCR1/NKp46, together with other NK-cell markers (see Fig 3) coincided with the presence of elevated IFN-γ transcript levels at week 5 in M7392, M7249, F7238, and F7386 (fold-change: 2.1–3.5). Liver tissues were also stained for IFN-γ for determining cytokine presence at the protein level, and the increases in the average mean and in the relative percentage of IFN-γ staining intensity correlated well with the increases in transcript level in all animals at this time (Fig 8 and S12 Fig). In blood (S13 Fig), IFN-γ expression started to increase during weeks 2–4 in all animals (fold-change: 2.1–3.1). Since the increase in intrahepatic IFN-γ at both the transcript and protein levels was observed around the peak, and then more so during the initial decline in WHV replication, this may indicate the presence of a non-cytolytic mechanism of viral clearance by NK-cells that mainly secrete this cytokine. Other cell subsets secreting IFN-γ include CTLs that were absent at this time based on unchanged CD3, CD8, and cytolytic effector molecule expression (see Fig 5 and Fig 6), and NKT-cells [26]. The latter cell subset was not tested in the present study. Following week 5, NCR1/NKp46 expression continued to increase and peaked during weeks 9–18 in all woodchucks, as also observed for other NK-cell markers (see Fig 3). CD3 expression became also elevated by week 9 and/or peaked during weeks 9–13 in most animals, as also noted for the expression of CD8 marker and cytolytic effector molecules (see Fig 5 and Fig 6). Serum SDH levels markedly rose starting at weeks 8–12 and/or were maximal elevated during weeks 8–18. Since the peak serum activity of SDH in individual woodchucks was temporally associated with the maximum expression of T-cell markers and cytolytic effector molecules, as well as with the marked decline in WHV replication, peak WHV-specific T-cell responses (see Fig 1), pronounced hepatitis/liver inflammation, and increased replenishment of liver with hepatocytes (Table 1), this may indicate the presence of a cytolytic mechanism of viral clearance by mainly CTLs. Furthermore, IFN-γ became predominantly expressed and maximal during weeks 9–13 in all animals (fold-change: 5.9–73.9). The relative percentage of intrahepatic IFN-γ staining also increased further and/or peaked during weeks 9–13. In blood (S13 Fig), peak IFN-γ expression was noted during weeks 10–12 in M7392, M7249, and F7238 (fold-change: 5.7–7.7). Based on the time points analyzed, IFN-γ transcript levels appeared maximal at week 4 for F7386 and at week 8 for F7394 (fold-change: 3.2–5.5). Overall, the kinetics of IFN-γ expression in liver and blood correlated well in most woodchucks. Since the peak IFN-γ transcription and secretion within liver was temporally associated with the maximum expression of NK-cell and CTL markers, this may suggest an overlap of non-cytolytic and cytolytic mechanisms of viral control by both immune cell subsets during this time. In addition, since the magnitude of intrahepatic IFN-γ expression was higher than the expression of all CTL markers tested (i.e., CD8, GZMB, PRF1, and FASL), this may indicate a dominance of the non-cytolytic over the cytolytic mechanism of WHV suppression in woodchucks.

## Discussion

Experimental infection of naïve, adult woodchucks with an equal dose of WHV resulted in acute, self-limiting infection in all five animals, which eventually resolved [17]. WHV rc-DNA was detected in serum as early as 1–2 weeks post-inoculation; this was followed by the appearance of WHsAg and WHeAg. These three serum markers reached peak levels thereafter, and then markedly declined. Comparable to studies in patients with AHB, viral surface antigen was present in all woodchucks before e antigen became detectable [27]. Correlating with the decline in antigemia, anti-WHs antibodies were first elicited, followed by anti-WHe antibodies, and the humoral response remained present until the end of the study. It has been reported for patients with AHB that the peak in ALT level is observed during the rapid decline of HBV DNA in serum [8,28]. Similarly, serum SDH (and AST) activity in all woodchucks was nearly unchanged at the time of peak viremia and antigenemia, indicating the non-cytopathic nature of WHV, but increased later, when a marked decline in viral replication was noted, suggesting acute liver injury (i.e., AHB) in these animals. This was associated with pronounced hepatic inflammation, increased replenishment of liver with new hepatocytes, and peripheral WHV-specific T-cell responses. The kinetics of WHV replication markers in liver, including RI-DNA, cccDNA, and pgRNA, followed a similar pattern of increase and subsequent decline, as observed for serum WHV rc-DNA [17]. Initial and then marked inhibition of WHV replication correlated with the expression of NK-cell markers and IFN-γ, followed by increases in the transcript levels of CTL markers, and subsequent maximum expression of both cell subsets, as well as cytolytic effector molecules and IFN-γ. Although there was individual variation in the course of acute, self-limited WHV infection, despite inoculation with the same WHV dose, as also observed in previous studies [12,29–31] the overall changes in viremia, antigenemia and AHB were similar between the five outbred animals, and thus comparable to those reported in patients [28].

For a detailed characterization of immune response involved in WHV resolution, selected innate and adaptive immune cell markers were determined in blood and liver by using the sequence information derived from the woodchuck transcriptome [32]. Previous studies in cell cultures and animal models, including woodchucks and chimpanzees, have shown that HBV and WHV apparently fail to induce an innate immune response during the early stages of infection, indicating a stealth-like behavior of hepadnaviruses [15,33–35]. In addition, only low serum levels of IFN-α were found in a study on early immune response in patients with AHB [8]. Consistent with these findings, almost all woodchucks of the present study did not display marked changes in the intrahepatic or peripheral expression of type I IFNs and ISGs during the initial 4–5 weeks post-inoculation, and despite already marked or even maximum viral replication in both compartments during this time.

An increased frequency of activated NK- and NKT-cells was observed during the early HBV incubation period in the periphery of patients who later developed AHB [9,27], and these cell subsets, likely including mucosal-associated invariant T-cells (MAITs) [10], may control HBV infection by producing IFN-γ. All woodchucks of the present study also exhibited markedly increased IFN-γ expression in liver and periphery, and elevated cytokine secretion in liver, during the initial and more so during the subsequent and pronounced reduction in viral replication. Furthermore, intrahepatic presence of IFN-γ at week 5 correlated with an expression increase of NK-cell activation receptors and markers, including NCR1/NKp46, KLRF1/NKp80, and HNK-1/CD57 in most animals, suggesting an important role of this immune cell subset in initially limiting WHV infection *via* IFN-γ. Although this cytokine can be secreted by other cell subsets, such as CTLs, increases in the intrahepatic expression of T-cell markers (i.e., CD3 and CD8) and cytolytic effector molecules (i.e., GZMB, PRF1, and FASL) and in the peripheral WHV-specific T-cell response were observed later at weeks 9 or 12, respectively. Since the increase in IFN-γ expression was noted prior to any elevation in liver enzyme activity and hepatitis/liver inflammation, this may indicate the presence of a non-cytolytic mechanism of initial viral control mediated by NK-cells. During WHV resolution, however, it was difficult to differentiate between immune cell subsets that secrete IFN-γ based on changes in transcript levels. Hence, an overlap in peak expression of T- and NK-cell markers and cytolytic effector molecules and IFN-γ, as well as maximum WHV-specific T-cell response, peak in liver enzyme activity, and pronounced hepatic inflammation and liver replenishment was observed during this time. This may indicate the presence of a cytolytic mechanism of later viral control mediated by CTLs and/or NK-cells.

Activated APCs release cytokines, such as IL-12 and IL-18, that stimulate IFN-γ and TNF-α production in NK-cells and MAITs, and promote intrahepatic HBV-specific T-cell expansion [10]. In this study, the intrahepatic peak expression of pDC and macrophage markers, and the maximum of infiltrating macrophages in liver, during weeks 9–18 coincided with increased and maximum NK- and T-cell transcript levels in all woodchucks.

The role of CD4+ and CD8+ T-cells in resolution of HBV infection has been extensively described [10]. The finding that a comparable adaptive immune response is absent in patients with CHB due to mainly T-cell anergy and exhaustion, only reasserts the importance of both immune cell subsets [23]. This has also been described for woodchucks where animals progressing to CHB have absent or incomplete WHV-specific T-cell responses, suggesting that the onset of chronic HBV infection in humans may be associated with deficiencies in the primary T-cell response to acute HBV infection [13]. Furthermore, this immune tolerance at the T-cell (and B-cell) level can be therapeutically reversed in woodchucks with established CHB *via* prolonged nucleoside treatment, and restored by WHsAg vaccination to an adaptive immune response profile comparable to that observed in WHV resolution [36]. Among the viral factors responsible for the cellular immune tolerance observed in CHB, maternal HBeAg has been implicated, since this soluble viral protein may be able to cross the placenta of HBV transgenic mice for inducing tolerance in utero, which subsequently manifests at the T-cell level [37]. After vertical transfer in transgenic mice, maternal HBeAg further mediates HBV persistence *via* macrophage-dependent suppression of CTL responses [38]. In support of these studies, experimental infection of neonatal woodchucks with an inoculum containing a precore WHeAg-minus mutation significantly reduced the chronic outcome of WHV infection [31].

In patients with AHB, a peak in peripheral T-cell response was observed, when serum HBV DNA started to decline and ALT level increased, suggesting a role of Th-cells and especially of CTLs in controlling the infection and leading to AHB [9,28]. Furthermore, a correlation between intrahepatic HBV-specific CD8+ T-cells and AHB was revealed in patients [39]. Mirroring these observations in humans, increased WHV-specific T-cell responses in blood, augmented expression of Th-cell and CTL markers in liver and blood, and increased infiltration of CD3+ and CD4+ in liver, correlated with the marked decline in WHV replication and elevation in liver enzyme activity in woodchucks. The effector function of woodchuck CTLs appeared to be mainly mediated by perforin and Fas receptor-ligand interaction in liver, but with dominant perforin and granzyme B expression in the periphery. NK-cells have been shown to also use cytotoxic granules, such as perforin and granzyme B, for their cytolytic effector function [26,40]. As mentioned above, the overlap in peak expression of CTL and NK-cell markers and cytolytic effector molecules, especially in liver, did not allow to determine if CTLs are primarily responsible for the cytolytic mechanism of viral clearance during WHV resolution.

The interaction of the PD-1 receptor with one of its ligands (i.e., PD-L1) has been shown to limit the function of CTLs *in vitro* by using blood cells from patients with AHB [41]. This interaction may be essential for balancing the cytopathic, antiviral function of the host immune response. The intrahepatic expression of these T_reg_ markers in woodchucks, together with PD-L2 and TGF-β, correlated with a subsequent dampening of liver injury, based on reduced levels of SDH in serum and of hepatitis/inflammation in liver. Upregulated T_reg_ marker expression has been observed previously in neonatal woodchucks which resolved AHB [15]. However, it is also possible that T_regs_ are only a part of the overall immune cell infiltration in liver and accumulate over time in this compartment.

Overall, the kinetics of innate and adaptive immune cell markers during acute, self-limited WHV infection suggest an initial control of viral replication by a non-cytolytic mechanism *via* IFN-γ produced primarily by NK-cells. The contribution of MAITs to this cytokine production was not investigated in the present study. This was followed by a mounting adaptive immune response, including antibodies produced by B-cells, WHV-specific T-cell responses, and a cytolytic mechanism likely mediated by CTLs, leading to the marked decline in WHV replication and to peak AHB. Of note is that apart from NK-cell markers and IFN-γ, there was no apparent correlation between the expression kinetics of other innate and adaptive immune cell markers in blood and liver of woodchucks, as determined by changes in transcript levels but not by functional assays. In addition, the expression of all markers tested was more pronounced in liver than in the periphery, which is not unexpected as the first compartment is the site of viral replication and disease. This further indicates the important role of hepatic resident and infiltrating immune cells and their enrichment within liver in regard to controlling hepadnavirus infection during acute, self-limited WHV and HBV infection.

From the results of this study in woodchucks, the immune response mounted against WHV infection is delayed, but complex and coordinated, and warrants further delineation for exploring new treatment options based on immunomodulation for patients with CHB in this animal model. Antiviral efficacy has been shown for treatment with an antibody against PD-L1, interferon-α, as well as with agonists of innate immune receptors in animal models of HBV [42,43]. The intrahepatic presence of NK-cell associated IFN-γ response 5 weeks after WHV infection indicates an involvement of this cytokine in the initial control of viral replication. Immunomodulation *via* the activation of toll-like receptor 8, therefore, appears a promising approach for inducing IFN-γ secretion in liver by NK-cells and MAITs [42,44,45]. Since the knowledge on immune response during the early stages of HBV infection and subsequent resolution in patients is most often restricted to the periphery, mainly due to the asymptomatic nature of disease onset and the lack of liver biopsies, future studies in immunocompetent animal models of HBV can address these limitations. The present study, however, emphasizes the resemblance of innate and adaptive immune responses associated with WHV resolution in woodchucks with those obtained during acute, self-limited HBV infection in humans.

## Materials and methods

### Ethics statement

The animal protocol entitled “Super-infection and virus spread during chronic hepadnaviral infection” and all procedures involving woodchucks were approved by the IACUC of Northeastern Wildlife, Inc. (Harrison, ID) on January 3, 2012, and adhered to the national guidelines of the NIH Guide for the Care and Use of Laboratory Animals. Woodchuck were anesthetized by intramuscular injection of ketamine (50 mg/kg) and xylazine (5 mg/kg) for blood collection and percutaneous liver biopsy. Prior to euthanasia, woodchucks were anesthetized as described above and euthanized by an overdose of pentobarbital (80–100 mg/kg) administered by intracardiac injection, followed by bilateral intercostal thoracotomy.

### Woodchucks and WHV infection

The five adult woodchucks investigated in the present study were part of a larger animal cohort that was experimentally infected with 1.66x10^8^ GE of WHV strain 7 as described recently [17]. Following the inoculation, all woodchucks were monitored for approximately 18 weeks. Serum, blood, and liver samples were collected prior to inoculation (week -2) and again at the end of the study (week +18). In addition, serum was collected weekly, whole blood was obtained bi-weekly, and additional liver biopsies were taken at weeks +5, +9, and +13 post-inoculation.

### Serum WHV and liver enzyme parameters

WHV rc-DNA was extracted from serum and measured by quantitative PCR [17]. Serum levels of WHeAg and anti-WHe antibodies were determined using cross-reactive ELISA assays (DiaSorin, Minneapolis, MN). Results were obtained as an optical density (OD) read out. An OD value of ≥0.047 indicated presence of WHeAg, while an OD value of ≥0.767 indicated presence of anti-WHe antibodies. Serum activity (IU/L) of AST, SDH, and GGT was determined in samples collected at weeks 0, +4, +8, +12, and +18.

### Hepatic WHV parameters

Liver samples were examined for sinusoidal and portal hepatitis/inflammation. The composite hepatitis score was obtained using a 0–6 scale, where 0 = absent, >0–2 = mild, >2–4 = moderate, and >4 = marked hepatitis/inflammation. Serum activity of AST, SDH, and GGT was used as markers of hepatocellular injury [46]. Hepatocyte proliferation was assayed by staining for the cellular proliferation marker, Ki67, with a cross-reactive antibody (Dianova, Hamburg, Germany). Liver tissues were further stained with an antibody to WHsAg [29]. Using a brightfield microscope and a 40-fold magnification, ten random liver sections were photographed, images digitally stored, stained and unstained hepatocytes identified, and the average percentage of cells expressing cytWHs obtained by manual count.

### WHV-specific T-cell response

Peripheral blood mononuclear cells (PBMCs) were isolated using the Ficoll-Paque density gradient centrifugation method and cultured in complete AIM-V medium (Thermo Fisher Scientific, Waltham, MA) in 96-well opaque plates (Sigma, St. Louis, MO) as described previously [36]. PBMCs were stimulated with 0.02% dimethyl sulfoxide (DMSO, Sigma, unstimulated negative control), 0.5 μg/mL lipopolysaccharide (LPS, Sigma, positive control), and pools of peptides covering the entire WHcAg or WHsAg (Thermo Fisher Scientific). Peptides were dissolved for obtaining a final concentration of 10.0 μg/mL for each peptide in 0.02% DMSO. After 5 days of stimulation, cell proliferation was determined with the CellTiter Glo One Solution assay (Promega, Madison, WI). The derived luminescence signal of triplicate cell cultures was averaged and expressed as a fold-change by dividing the average signal in the presence of stimulator (WHcAg- or WHsAg-derived peptides) by that in the absence of stimulator (DMSO-containing medium) at a particular week. A fold-change of ≥2.1 from control cultures was considered a positive result for the presence of increased WHV-specific T-cell response.

### Peripheral and hepatic immune response parameters

Whole blood was collected into PAXgene blood tubes (Qiagen, Redwood City, CA) and stored at -80°C until use. Total RNA was isolated with on-column DNase I digestion using the PAXgene Blood miRNA kit (Qiagen). Total RNA was further isolated from liver tissue using the RNeasy Mini kit (Qiagen) with on-column DNase I digestion using RNase-free DNase (Qiagen). Following reverse transcription of mRNA with the High Capacity cDNA Reverse Transcription kit (Applied Biosystems, Foster City, CA) using oligo(dT), complementary (c) DNA samples were amplified on a 7500 Real Time PCR System instrument (Applied Biosystems) using TaqMan or SYBER Green Gene Expression Master Mix (Applied Biosystems). Target genes investigated (S1 Table) included markers for: (i) type I IFNs and ISGs; (ii) NK-cells; (iii) APCs; (iv) Th-cells; (v) CTLs; and (vi) T_regs_. The woodchuck-specific primers and probes are presented in S2 Table. 18S rRNA expression was used to normalize target gene expression. Transcript levels of target genes were calculated as a fold-change relative to the pre-inoculation (baseline) level in blood and liver at week -2 (or at week 0 prior to inoculation in blood of woodchuck F7394 and woodchucks M7392 and M7249 for selected genes) using the formula 2^-ΔΔ*Ct*^. A fold-change of ≥2.1 from this baseline was considered a positive result for the presence of increased target gene expression.

### Immunohistochemistry staining

Liver tissues were stained with cross-reactive antibodies to markers of cell subsets and cytokines, including CD3 (Novocastra/Leica Biosystems, Richmond, IL), CD4 (Abcam, Cambridge, MA), macrophage (LifeSpan BioSciences, Seattle, WA), and IFN-γ (Invitrogen, Carlsbad, CA). Ten random sections of each liver tissue were photographed at a 40-fold magnification using a brightfield microscopy equipped with digital camera and images digitally stored. The average percentages of stained cells were obtained by manual count, except for IFN-γ, where the average mean intensity of staining was measured using the ImageJ software (National Institutes of Health, Bethesda, MD). The maximum average mean staining intensity in a set of liver tissues collected from an individual woodchuck was set at 100% and the relative percentages from other tissues were subsequently calculated.

## Supporting information

S1 Table Genes investigated for analysis of innate and adaptive immune responses in woodchuck blood and liver. (DOCX)

S2 Table Oligonucleotides used for analysis of innate and adaptive immune responses in woodchuck blood and liver. (DOCX)

S1 Fig Percentages of WHsAg-positive cells in liver. Liver tissues of woodchucks collected at the indicated weeks before and after WHV inoculation were stained with an antibody to WHsAg. One representative image is shown for each timepoint. The percentages of cells positive for cytoplasmic WHsAg expression were obtained as described in the Materials and Methods and are provided below each image.
(TIF)

S2 Fig Percentages of Ki67-positive cells in liver. Liver tissues of woodchucks collected at the indicated weeks before and after WHV inoculation were stained with a cross-reactive antibody to Ki67. One representative image is shown for each timepoint. The percentages of Ki67-positive cells are provided below each image.
(TIF)

S3 Fig Peripheral expression of type I IFNs and ISGs. Changes in the expression of IFN-α, IFN-β, OAS1, and viperin in the periphery. The fold-change in transcript level of genes from baseline is plotted on the right y-axis, while serum WHV rc-DNA loads are plotted on the left y-axis.
(TIF)

S4 Fig Intrahepatic and peripheral expression of NK-cell receptors and surface markers. (**A**) Changes in the expression of KLRK1/NKG2D, KLRC1/NKG2A, and CD16 in the liver. (**B**) Changes in the expression of KLRK1/NKG2D, KLRC1/NKG2A, and CD16 in the periphery. In (A) and (B), the fold-change in transcript level of genes from baseline is plotted on the right y-axis, while serum WHV rc-DNA loads are plotted on the left y-axis.
(TIF)

S5 Fig Percentages of macrophages in liver. Liver tissues of woodchucks collected at the indicated weeks before and after WHV inoculation were stained with a cross-reactive antibody to MAC2, a macrophage marker. One representative image is shown for each timepoint. The percentages of MAC2-positive cells are provided below each image.
(TIF)

S6 Fig Peripheral expression of APC markers. Changes in the expression of CD79B (B-cell), IL3RA/CD123 (pDC), and EMR1/F4/80 (macrophage) in the periphery. The fold-change in transcript level of genes from baseline is plotted on the right y-axis, while serum WHV rc-DNA loads are plotted on the left y-axis.
(TIF)

S7 Fig Percentages of CD3-positive cells in liver. Liver tissues of woodchucks collected at the indicated weeks before and after WHV inoculation were stained with a cross-reactive antibody to CD3. One representative image is shown for each timepoint. The percentages of CD3-positive cells are provided below each image.
(TIF)

S8 Fig Percentages of CD4-positive cells in liver. Liver tissues of woodchucks collected at the indicated weeks before and after WHV inoculation were stained with a cross-reactive antibody to CD4. One representative image is shown for each timepoint. The percentages of CD4-positive cells are provided below each image.
(TIF)

S9 Fig Peripheral expression of T-cell markers. Changes in the expression of CD3, CD4, and CD8 in the periphery. The fold-change in transcript level of genes from baseline is plotted on the right y-axis, while serum WHV rc-DNA loads are plotted on the left y-axis.
(TIF)

S10 Fig Peripheral expression of markers for CD8+ T-cells and cytolytic effector molecules. Changes in the expression of CD8, GZMB, PRF1, and FASL in the periphery. The fold-change in transcript level of genes from baseline is plotted on the right y-axis, while serum WHV rc-DNA loads are plotted on the left y-axis.
(TIF)

S11 Fig Peripheral expression of T_reg_ markers. Changes in the expression of TGF-β, PD-1, PD-L1, and PD-L2 in the periphery. The fold-change in transcript level of genes from baseline is plotted on the right y-axis, while serum WHV rc-DNA loads are plotted on the left y-axis.
(TIF)

S12 Fig Mean intensities of IFN-γ staining of cells in liver. Liver tissues of woodchucks collected at the indicated weeks before and after WHV inoculation were stained with a cross-reactive antibody to IFN-γ. One representative image is shown for each timepoint. The average mean intensity of IFN-γ staining and the relative percentages of staining intensity are provided below each image. The maximum of average mean staining intensity is indicated by an asterisk.
(TIF)

S13 Fig Peripheral expression of IFN-γ. The fold-change in blood transcript level of IFN-γ from baseline is plotted on the right y-axis, while serum WHV rc-DNA loads are plotted on the left y-axis.
(TIF)
