# Supplementary material for: Innate and adaptive immunity associated with resolution of acute woodchuck hepatitis virus infection in adult woodchucks
Source: PLoS Pathog. 2019 Dec 23;15(12):e1008248. doi: 10.1371/journal.ppat.1008248 (PMC6946171; doi:10.1371/journal.ppat.1008248)
Supplement: S1 Table — (DOCX) [file ppat.1008248.s001.docx]

**S1 Table. Genes investigated for analysis of innate and adaptive immune responses in woodchuck blood and liver.**

| Marker Group | Genes |
| --- | --- |
| Type I IFNs | IFN-α = Interferon-alpha |
|  | IFN-β = Interferon-beta |
| ISGs | OAS1 = 2’-5’- oligoadenylate synthetase 1 |
|  | Viperin (RSAD2) = Radical S-adenosyl methionine containing domain protein 2 |
| NK-cells | NCR1/NKp46 = Natural cytotoxicity triggering receptor 1 |
|  | NCAM/CD56 = Neural cell adhesion molecule |
|  | KLRF1/NKp80 = Killer cell lectin-like receptor F1 |
|  | IFN-γ = Interferon-gamma |
|  | KLRK1/NKG2D = Killer cell lectin-like receptor K1 |
|  | KLRC1/NKG2A = Killer cell lectin-like receptor C1 |
|  | CD57 (HNK-1) = Human natural killer-1 receptor |
|  | CD16 = Cluster of differentiation 16 |
| APCs | CD79B = Cluster of differentiation 79B, B-cell marker |
|  | IL3RA/CD123 = Interleukin 3 receptor A or CD123, plasmacytoid dendritic cell marker |
|  | EMR1/ F4/80 = EGF-like module-containing mucin-like hormone receptor like-1, macrophage marker |
| Th-cells | CD3 = Cluster of differentiation 3, T-cell co-receptor |
|  | CD4 = Cluster of differentiation 4, T-cell co-receptor |
| CTLs | CD8 = Cluster of differentiation 8, T-cell co-receptor |
|  | GZMB = Granzyme B or cytotoxic T-lymphocyte-associated serine esterase |
|  | PRF1 = Perforin |
|  | FASL = Fas ligand |
| Tregs | TGF-β = Transforming growth factor-beta |
|  | PD-1 = Programmed cell death-1 receptor |
|  | PD-L1 = Programmed cell death-1 ligand 1 or CD274 |
|  | PD-L2 = Programmed cell death-1 ligand 2 or CD273 |
