# Supplementary material for: Innate and adaptive immunity associated with resolution of acute woodchuck hepatitis virus infection in adult woodchucks
Source: PLoS Pathog. 2019 Dec 23;15(12):e1008248. doi: 10.1371/journal.ppat.1008248 (PMC6946171; doi:10.1371/journal.ppat.1008248)
Supplement: S2 Table — (DOCX) [file ppat.1008248.s002.docx]

**S2 Table. Oligonucleotides used for analysis of innate and adaptive immune responses in woodchuck blood and liver.**

| Gene | Primers and Probe | Sequence |
| --- | --- | --- |
| 18S rRNA | F | 5’- GTAACCCGTTGAACCCCATT-3’ |
|  | R | 5’- GGGACTTAATCAACGCAAGC-3’ |
|  | P | 5’- GCAATTATTCCCCATGAACG-3’ |
| IFN-α | F | 5’- CTCAAGCTGTTGCTGTCCTC -3’ |
|  | R | 5’- CTTCTGGGTGCTGAAGAGGT -3’ |
|  | P | 5’- CCAGATGACCCAGCAGATCCTCA -3’ |
| IFN-β | F | 5’- GAATGAAAGGCCTGCAGAGT -3’ |
|  | R | 5’- GAATGTTTGATCTCCTTGGG -3’ |
|  | P | 5’- CTTGAAGTCCATCCTGTCACTGAGGC -3’ |
| OAS1 | F | 5’- GACCCAGCCCCCAGATCT -3’ |
|  | R | 5’ -AACTCGCCCTCTAGCTGCC -3’ |
|  | P | 5’ -TGTCAAGCTCATCGAGGAGTGCACCT -3’ |
| Viperin | F | 5’ -TCTCCACGTGGTTCTTCTTTCCTT -3’ |
|  | R | 5’- TTGGACATTCTCGCCATTTCCTGT -3’ |
|  | P | 5’- ACGGCCAATAAGGACGTTGACTTCCT -3’ |
| NCR1/NKp46 | F | 5’- CTACAGATGCTTTGGCTCCTA -3’ |
|  | R | 5’- TGCAAGACTGCTGTTCCC -3’ |
| NCAM/CD56 | F | 5’- GCAGGAGATGCCAAAGATAA -3’ |
|  | R | 5’- TCATCGATATTGGCGTTGTAG -3’ |
| KLRF1/NKp80 | F | 5’- AGTCACAGGCCAGATTAAAGAG -3’ |
|  | R | 5’- GCCTGCTGTGGATGAAGAA -3’ |
| IFN-γ | F | 5’- ATCCAAAGGAGCATGGACAC -3’ |
|  | R | 5’- TGAACTTGAGACACCTTTAGGAA -3’ |
|  | P | 5’- CAACAGCAGTACCAATAAGCTGCAGGA -3’ |
| KLRK1/NKG2D | F | 5’- ACAGTAGAGTGGAACAGGATTT -3’ |
|  | R | 5’- GTTGGTTGGGCAAGAGAATG -3’ |
| KLRC1/NKG2A | F | 5’- CTGTCATGGGTTGGAGTCTTT -3’ |
|  | R | 5’- AGTACAGCATAGCACAGTTACG -3’ |
| HNK-1/CD57 | F | 5’- GTCAACCTGAGGCTCATTCTAC -3’ |
|  | R | 5’- TTTCCTGGTAGCCTCCTTTCACGC -3’ |
| CD16 | F | 5’- CTCTGTGTACAGCAACCTCATC -3’ |
|  | R | 5’- GGAAACAGAGAGTTGGGAGAAG -3 |
| CD79B | F | 5’- ACCCTCCTCATCATCCTCTT -3’ |
|  | R | 5’- CAATGTCCAGGCCCTCATAG -3’ |
|  | P | 5’- ATCGTGCCCATCTTCCTGTTGCT-3’ |
| IL3RA/CD123 | F | 5’- GTTCGTGGGAATCGTCTCAT -3’ |
|  | R | 5’- GAGGATGTTCAGCCACTTCA -3’ |
|  | P | 5’- AAGCTGCCTCCTGAGCTCTTGTTT -3’ |
| EMR1/F4/80 | F | 5’- AGTGCTTTCCTGCTTTCTCATA -3’ |
|  | R | 5’- GTGGAATCTTGCATCTCCATAG -3’ |
|  | P | 5’- AGAAGGGAAATCAGACCAGGCTCC -3’ |
| CD3 | F | 5’- CGGAGTTCGCCAGTCAAGA -3’ |
|  | R | 5’- TTGGTGGTTTCCTTGAAGACG -3’ |
|  | P | 5’ - CTTCAGACAAGCAGACTCTGTTGCCCAA - 3’ |
| CD4 | F | 5’- AGGTCTCAAAGCCCGAGAAGA -3’ |
|  | R | 5’- GTAGGCACTGCCACATCCCT -3’ |
|  | P | 5’ - ATTCGGGTGCCAAACCCCAAGG - 3’ |
| CD8 | F | 5’-TGGACTTCGCCTGTGATATCTACA -3’ |
|  | R | 5’- GTTTCCGGTGGTGACAGATGA -3’ |
|  | P | 5’ - TGCGCGGTCCTTCTGTTGTCACTG - 3’ |
| GZMB | F | 5’- TGACATCATGTTATTGGAGCTAG -3’ |
|  | R | 5’- CCCAGGCTTCACCTTGTCC -3’ |
|  | P | 5’- TGCAGCCTATCAAGCTGCCCAGG -3’ |
| PRF1 | F | 5’ – CCACCGAGCCTGACTACCTC -3’ |
|  | R | 5’ – CCAGCTCCACGGACCGGA -3’ |
|  | P | 5’ - CATCCACAACTACGGCA |
| FASL | F | 5’- ATCCCCAGGACCTGGTGC -3’ |
|  | R | 5’- GGCCCACATCTGGCCA -3’ |
|  | P | 5’- TGGAGGGCAAGATGATG -3’ |
| TGF-β | F | 5’-GGTAAAAGCCGAACAGCAC-3’ |
|  | R | 5’-CTCCTGCGTGTTACTGGG-3’ |
|  | P | 5’-AATTCCTGGCGCTACCTCAGCA-3’ |
| PD-1 | F | 5’- GGGTGACCTGGTTGCTG -3’ |
|  | R | 5’- CAGCTGAAGAGGCTTGAGG -3’ |
|  | P | 5’- CCTGTTGGAGGACTCTAGGAGCCATC -3’ |
| PD-L1 | F | 5’- ACAGCTGAATTGGTCATCCC -3’ |
|  | R | 5’- CACCAAAGCAAAACAGGACAG -3’ |
|  | P | 5’ -AAAGGACTCACTTTGTGAAGCTGGGA -3’ |
| PD-L2 | F | 5’- GGACTACAAGTACCTGACTGTG -3’ |
|  | R | 5’- ATGTGAGTTCCACCTCATCTG -3’ |
|  | P | 5’- CCTGTGACCTTGAGGATGCCAGT -3’ |

F: forward primer; R: reverse primer; P: probe.
