# Supplementary figures and images for: Innate and adaptive immunity associated with resolution of acute woodchuck hepatitis virus infection in adult woodchucks
Source: PLoS Pathog. 2019 Dec 23;15(12):e1008248. doi: 10.1371/journal.ppat.1008248 (PMC6946171; doi:10.1371/journal.ppat.1008248)

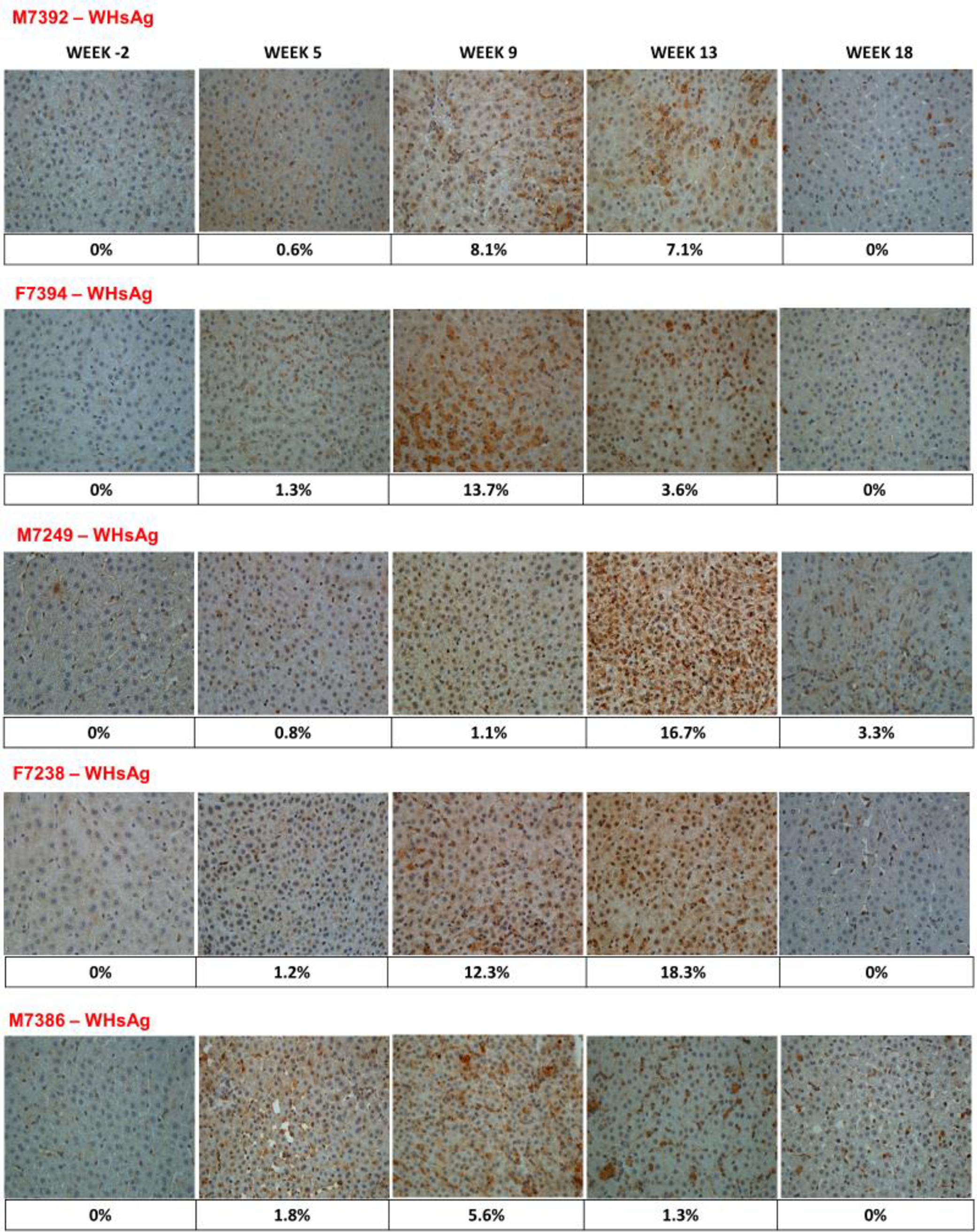

Supplement: S1 Fig — Liver tissues of woodchucks collected at the indicated weeks before and after WHV inoculation were stained with an antibody to WHsAg. One representative image is shown for each timepoint. The percentages of cells positive for cytoplasmic WHsAg expression were obtained as described in the Materials and Methods and are provided below each image. (TIF) [file ppat.1008248.s003.tif]

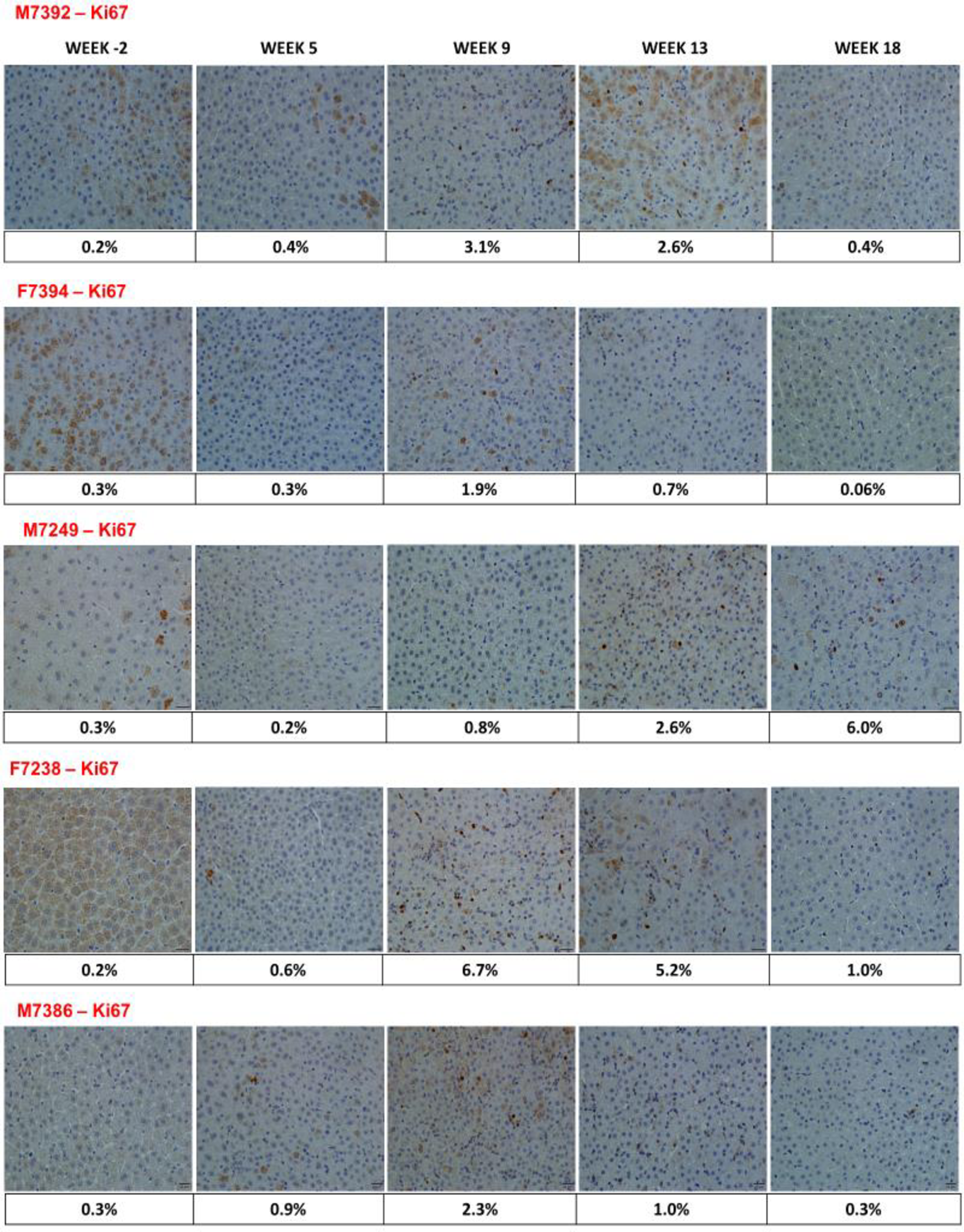

Supplement: S2 Fig — Liver tissues of woodchucks collected at the indicated weeks before and after WHV inoculation were stained with a cross-reactive antibody to Ki67. One representative image is shown for each timepoint. The percentages of Ki67-positive cells are provided below each image. (TIF) [file ppat.1008248.s004.tif]

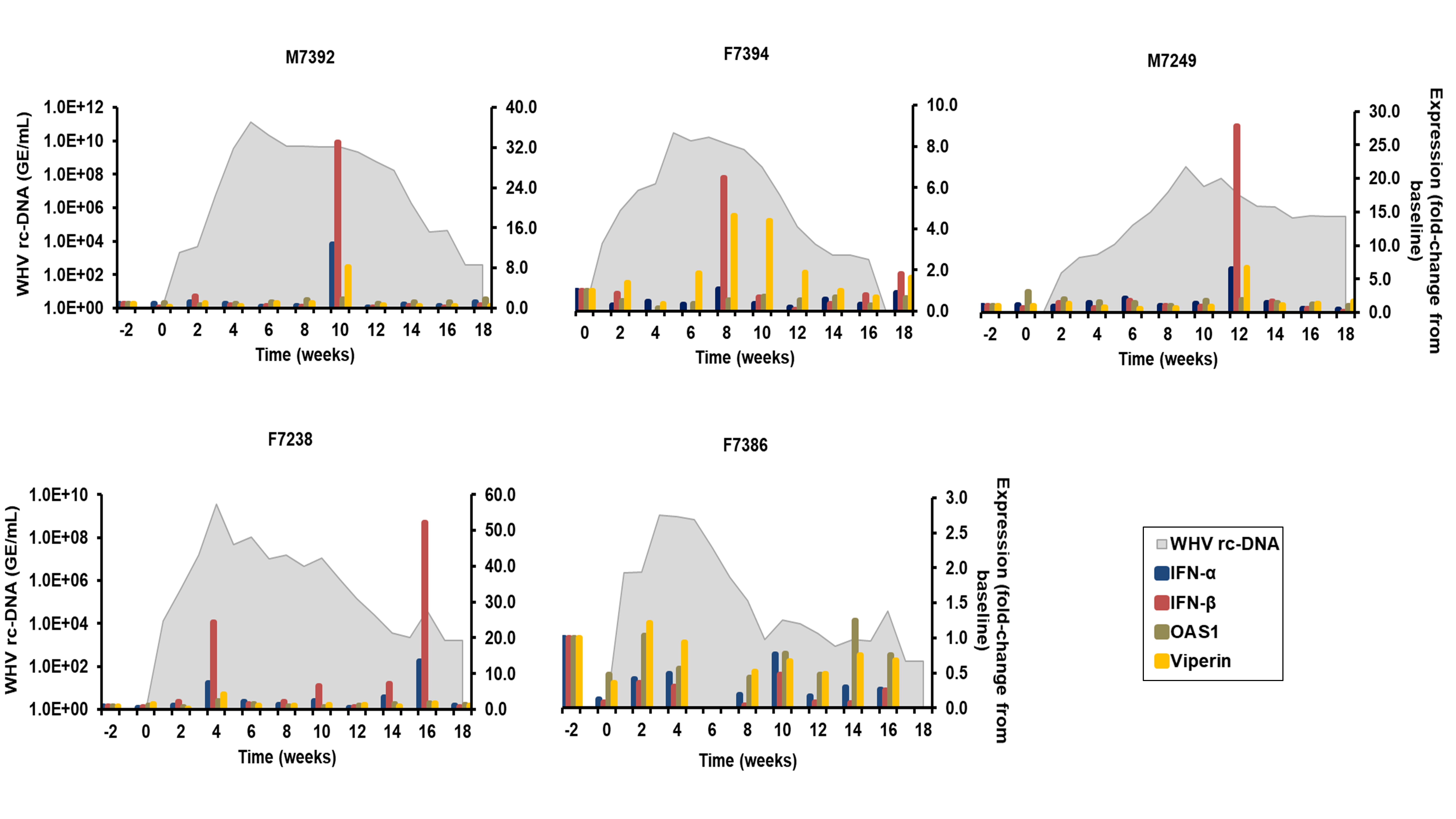

Supplement: S3 Fig — Changes in the expression of IFN-α, IFN-β, OAS1, and viperin in the periphery. The fold-change in transcript level of genes from baseline is plotted on the right y-axis, while serum WHV rc-DNA loads are plotted on the left y-axis. (TIF) [file ppat.1008248.s005.tif]

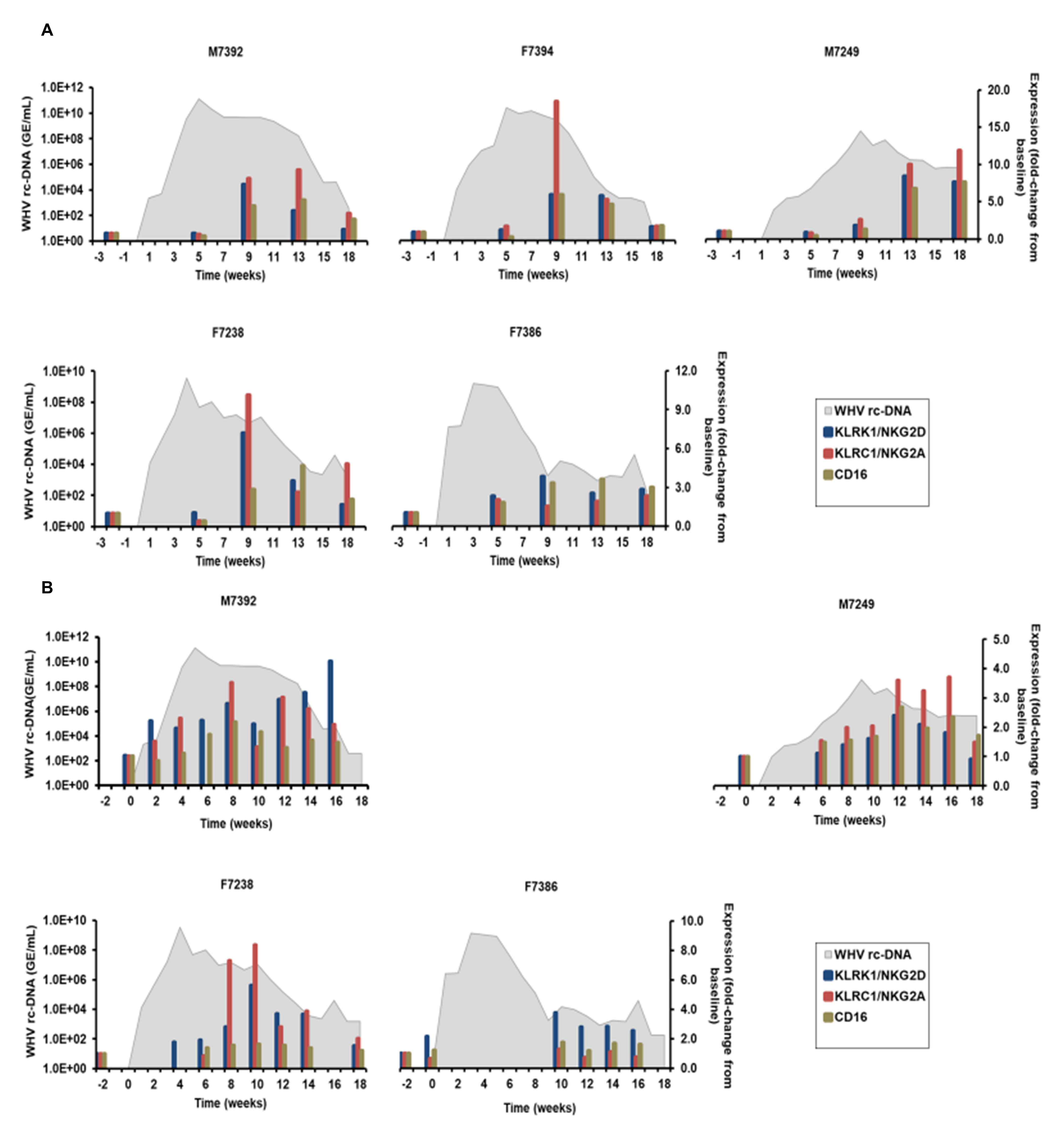

Supplement: S4 Fig — (A) Changes in the expression of KLRK1/NKG2D, KLRC1/NKG2A, and CD16 in the liver. (B) Changes in the expression of KLRK1/NKG2D, KLRC1/NKG2A, and CD16 in the periphery. In (A) and (B), the fold-change in transcript level of genes from baseline is plotted on the right y-axis, while serum WHV rc-DNA loads are plotted on the left y-axis. (TIF) [file ppat.1008248.s006.tif]

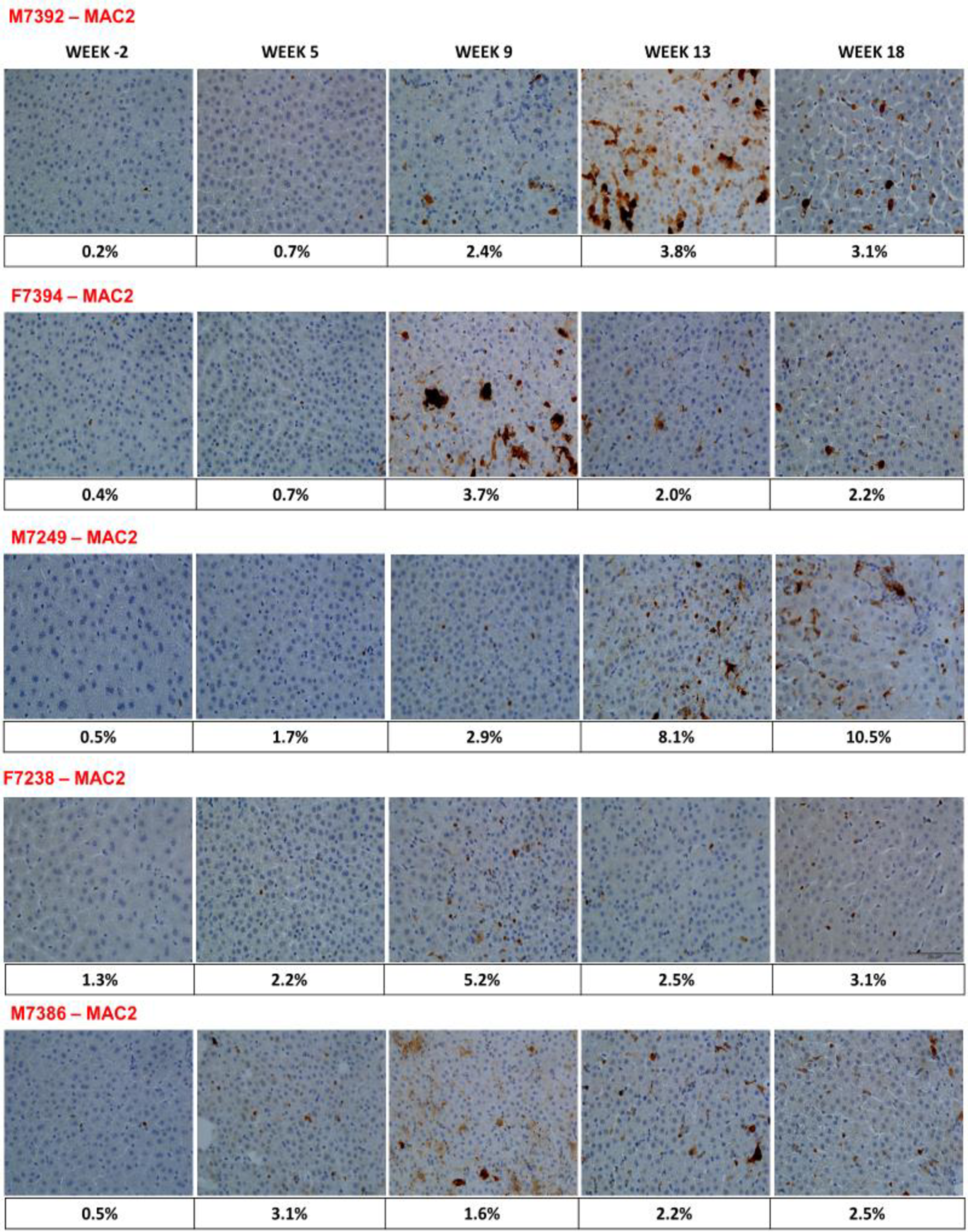

Supplement: S5 Fig — Liver tissues of woodchucks collected at the indicated weeks before and after WHV inoculation were stained with a cross-reactive antibody to MAC2, a macrophage marker. One representative image is shown for each timepoint. The percentages of MAC2-positive cells are provided below each image. (TIF) [file ppat.1008248.s007.tif]

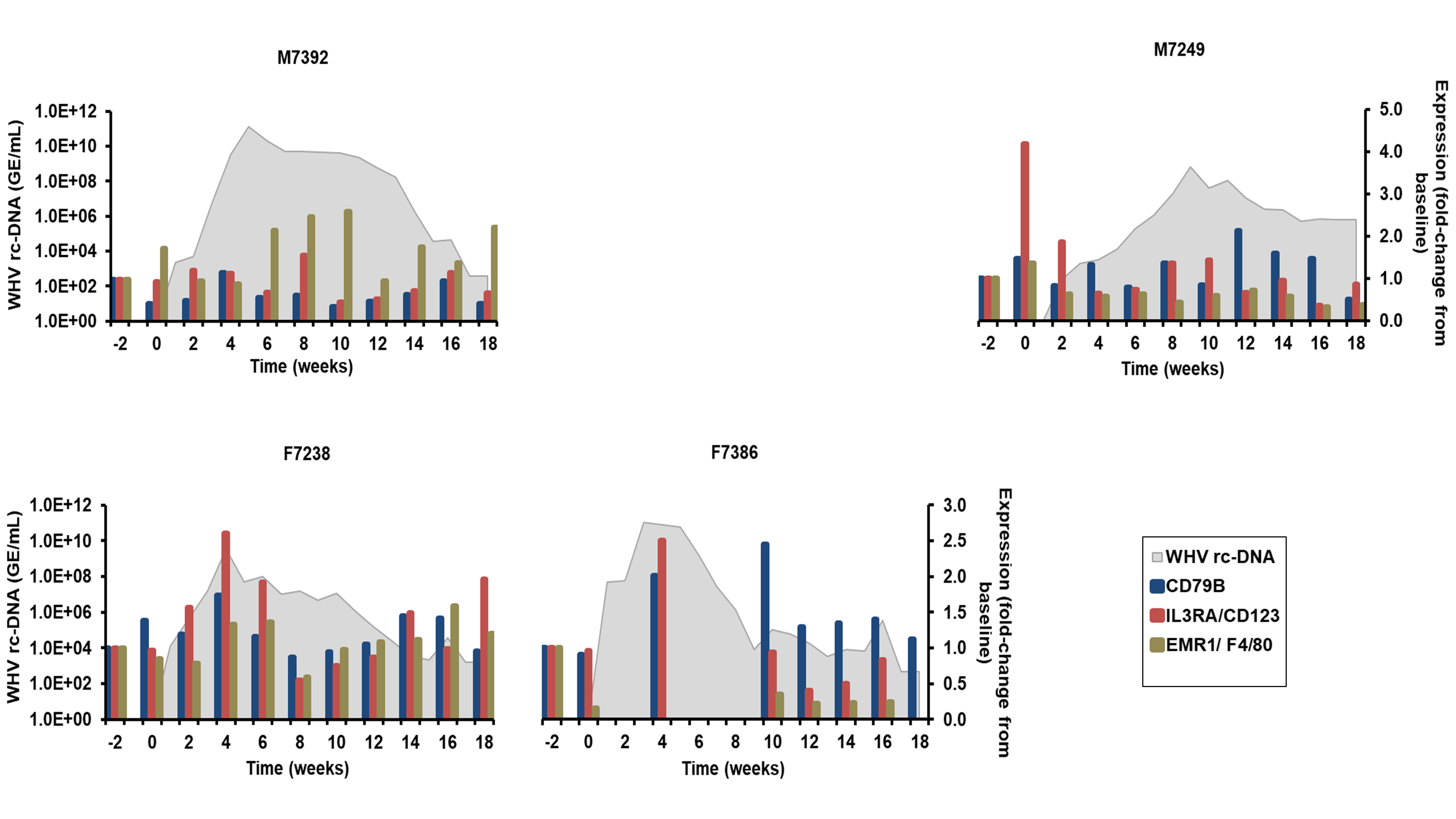

Supplement: S6 Fig — Changes in the expression of CD79B (B-cell), IL3RA/CD123 (pDC), and EMR1/F4/80 (macrophage) in the periphery. The fold-change in transcript level of genes from baseline is plotted on the right y-axis, while serum WHV rc-DNA loads are plotted on the left y-axis. (TIF) [file ppat.1008248.s008.tif]

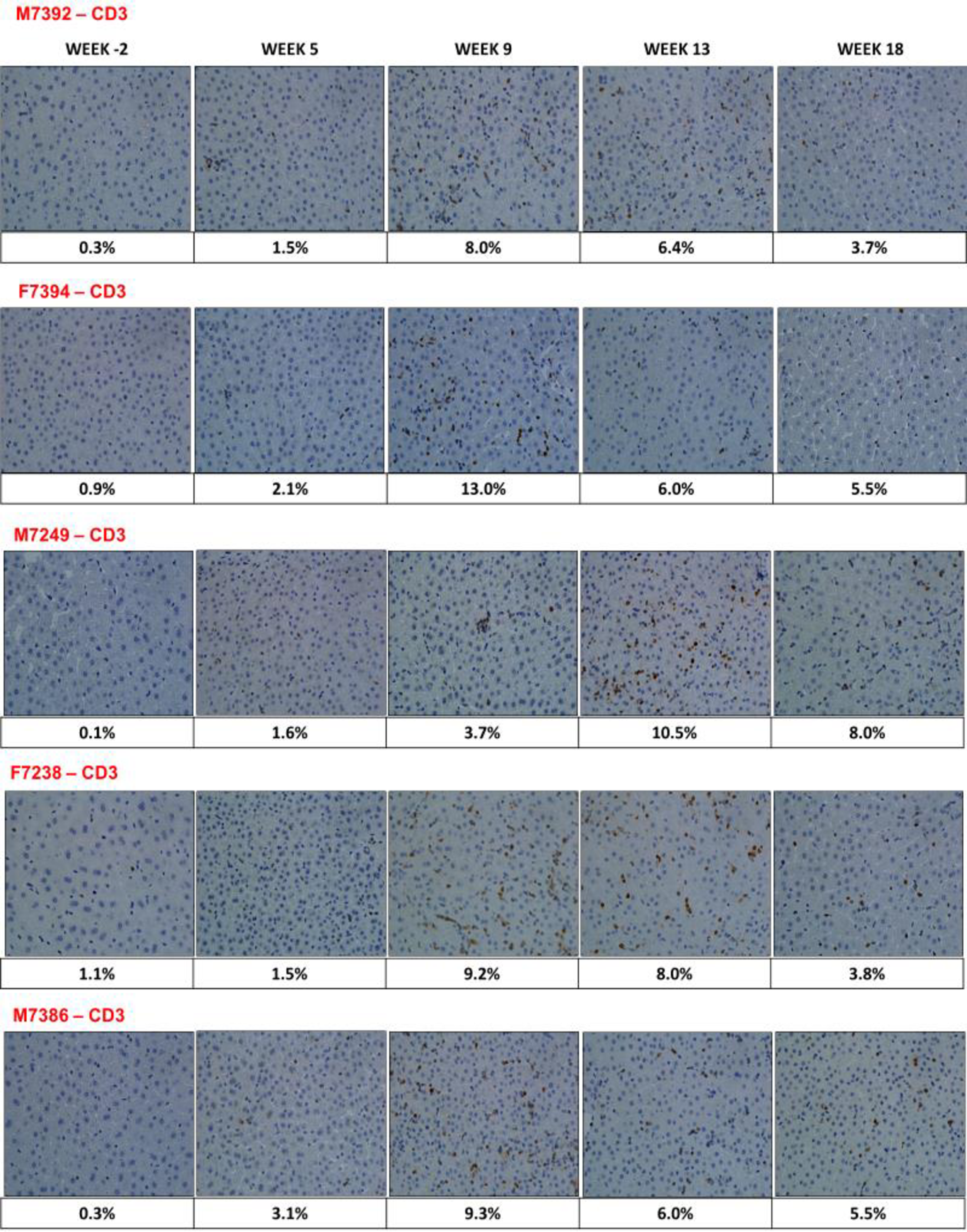

Supplement: S7 Fig — Liver tissues of woodchucks collected at the indicated weeks before and after WHV inoculation were stained with a cross-reactive antibody to CD3. One representative image is shown for each timepoint. The percentages of CD3-positive cells are provided below each image. (TIF) [file ppat.1008248.s009.tif]

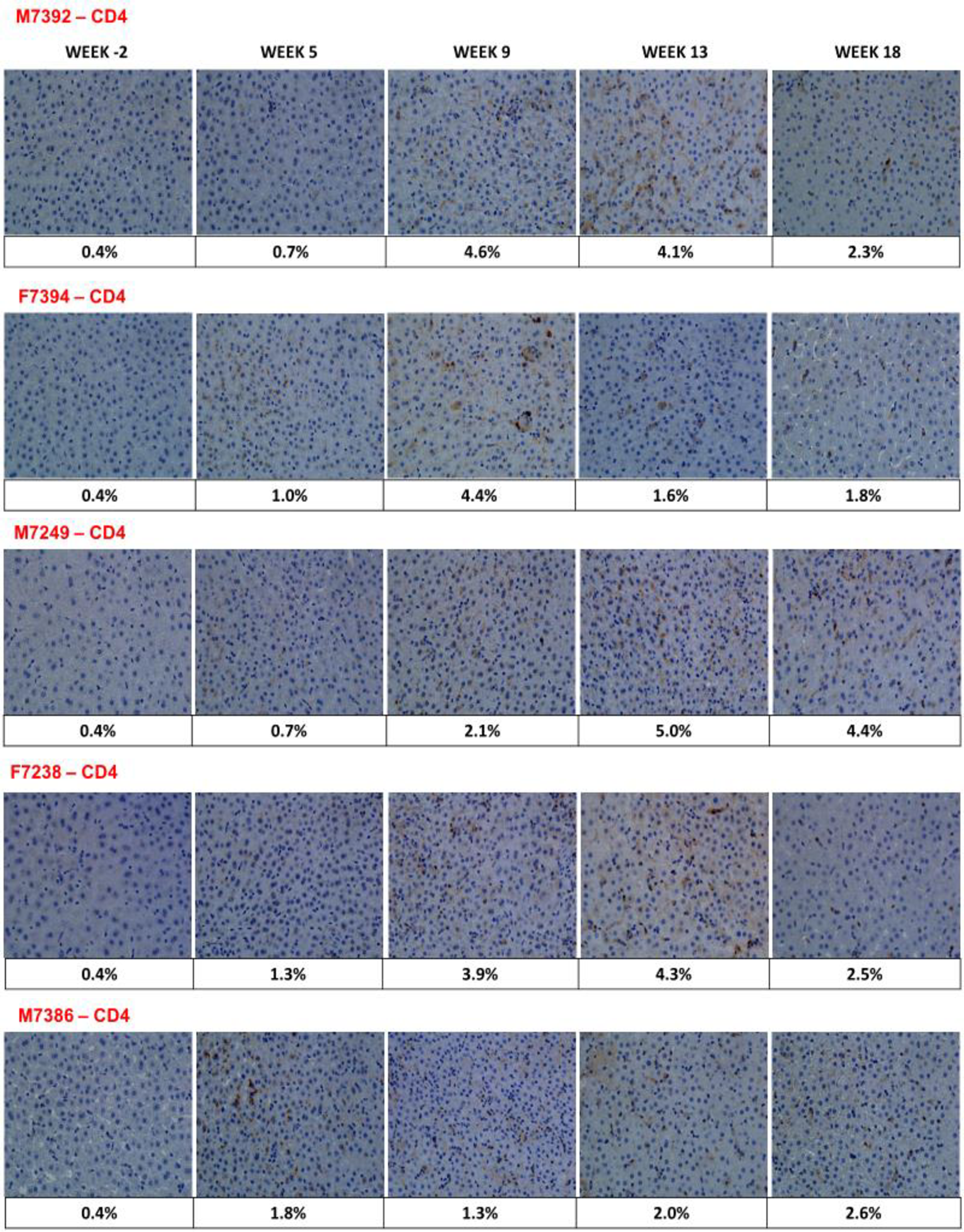

Supplement: S8 Fig — Liver tissues of woodchucks collected at the indicated weeks before and after WHV inoculation were stained with a cross-reactive antibody to CD4. One representative image is shown for each timepoint. The percentages of CD4-positive cells are provided below each image. (TIF) [file ppat.1008248.s010.tif]

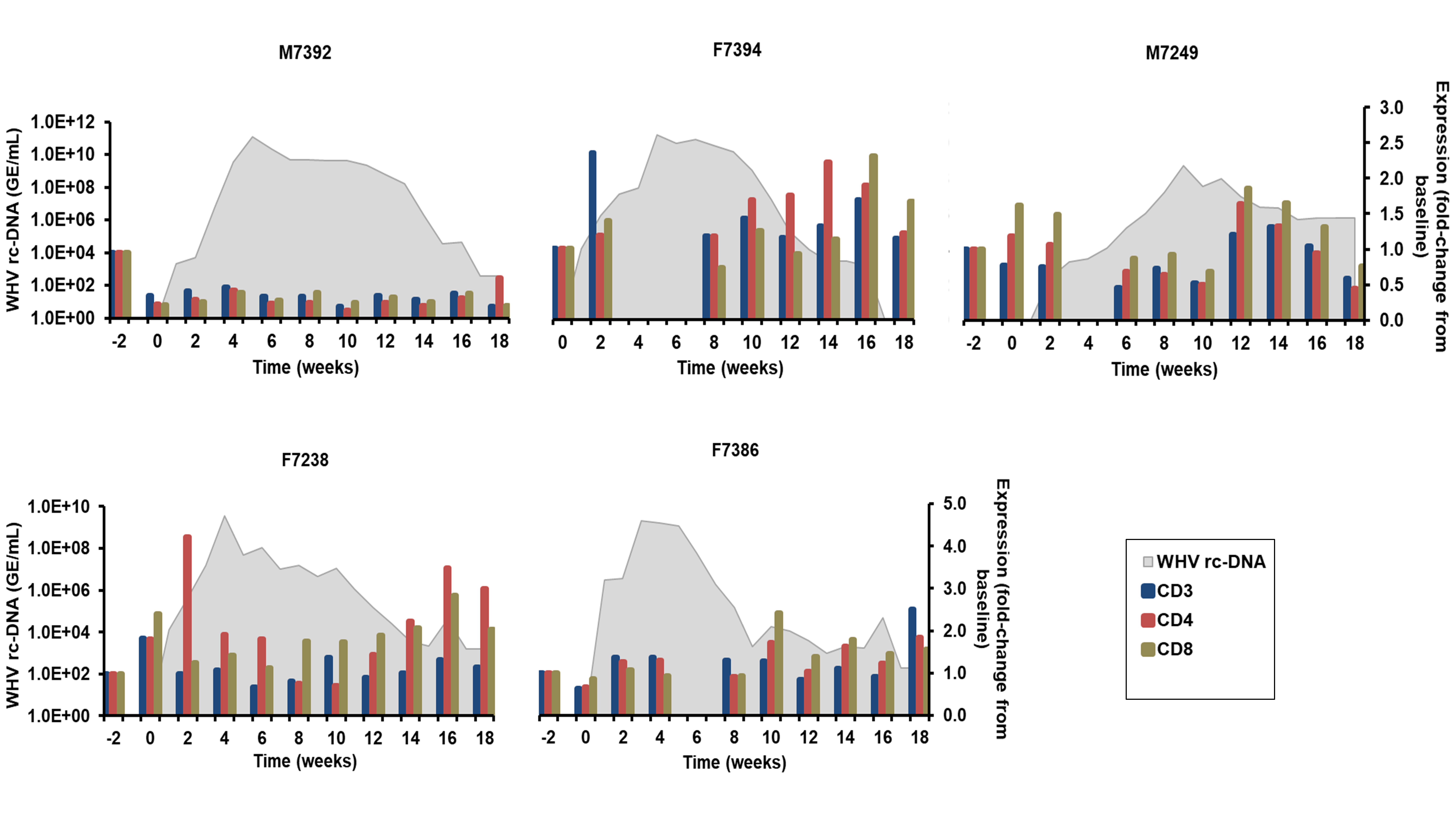

Supplement: S9 Fig — Changes in the expression of CD3, CD4, and CD8 in the periphery. The fold-change in transcript level of genes from baseline is plotted on the right y-axis, while serum WHV rc-DNA loads are plotted on the left y-axis. (TIF) [file ppat.1008248.s011.tif]

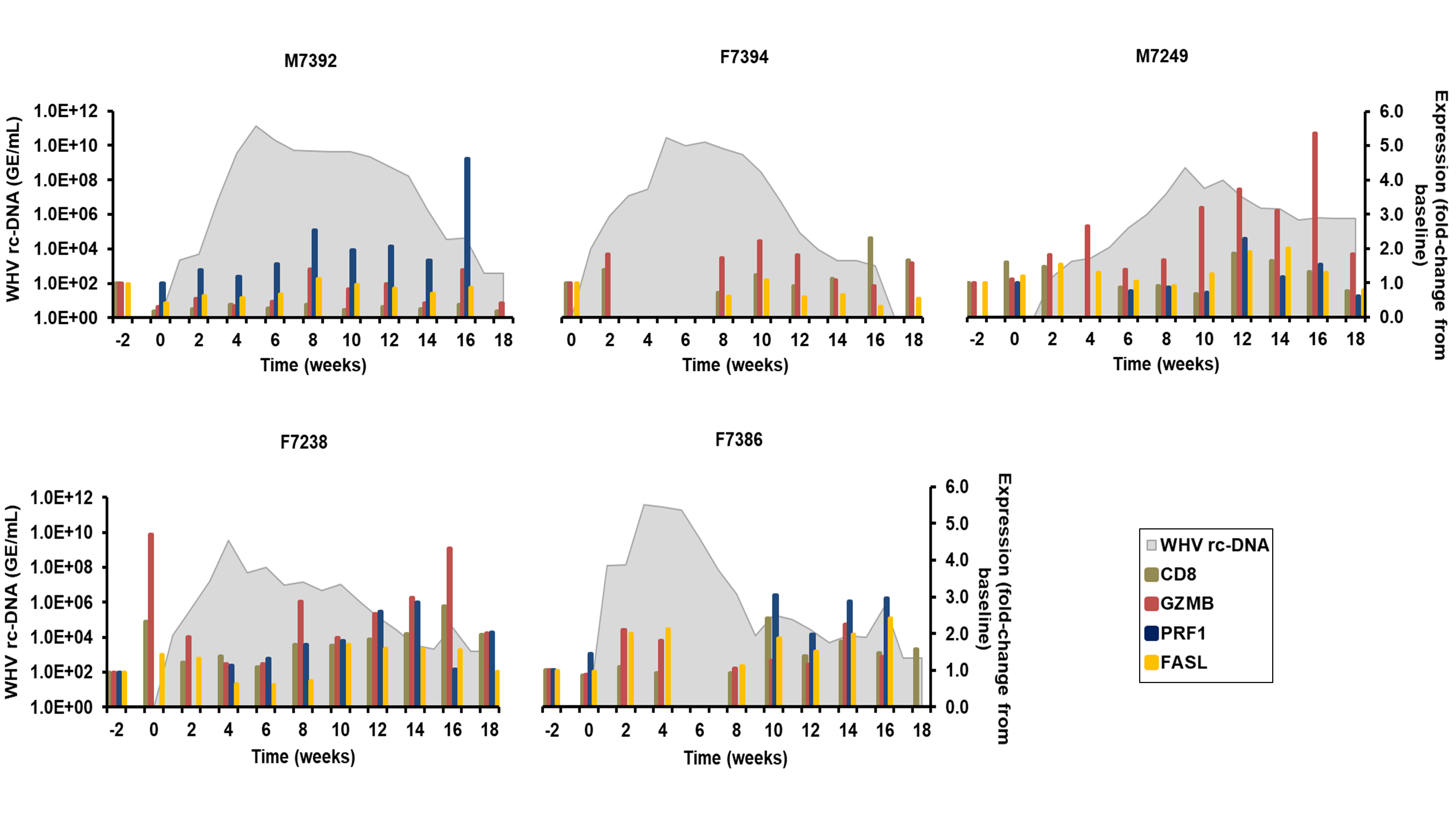

Supplement: S10 Fig — Changes in the expression of CD8, GZMB, PRF1, and FASL in the periphery. The fold-change in transcript level of genes from baseline is plotted on the right y-axis, while serum WHV rc-DNA loads are plotted on the left y-axis. (TIF) [file ppat.1008248.s012.tif]

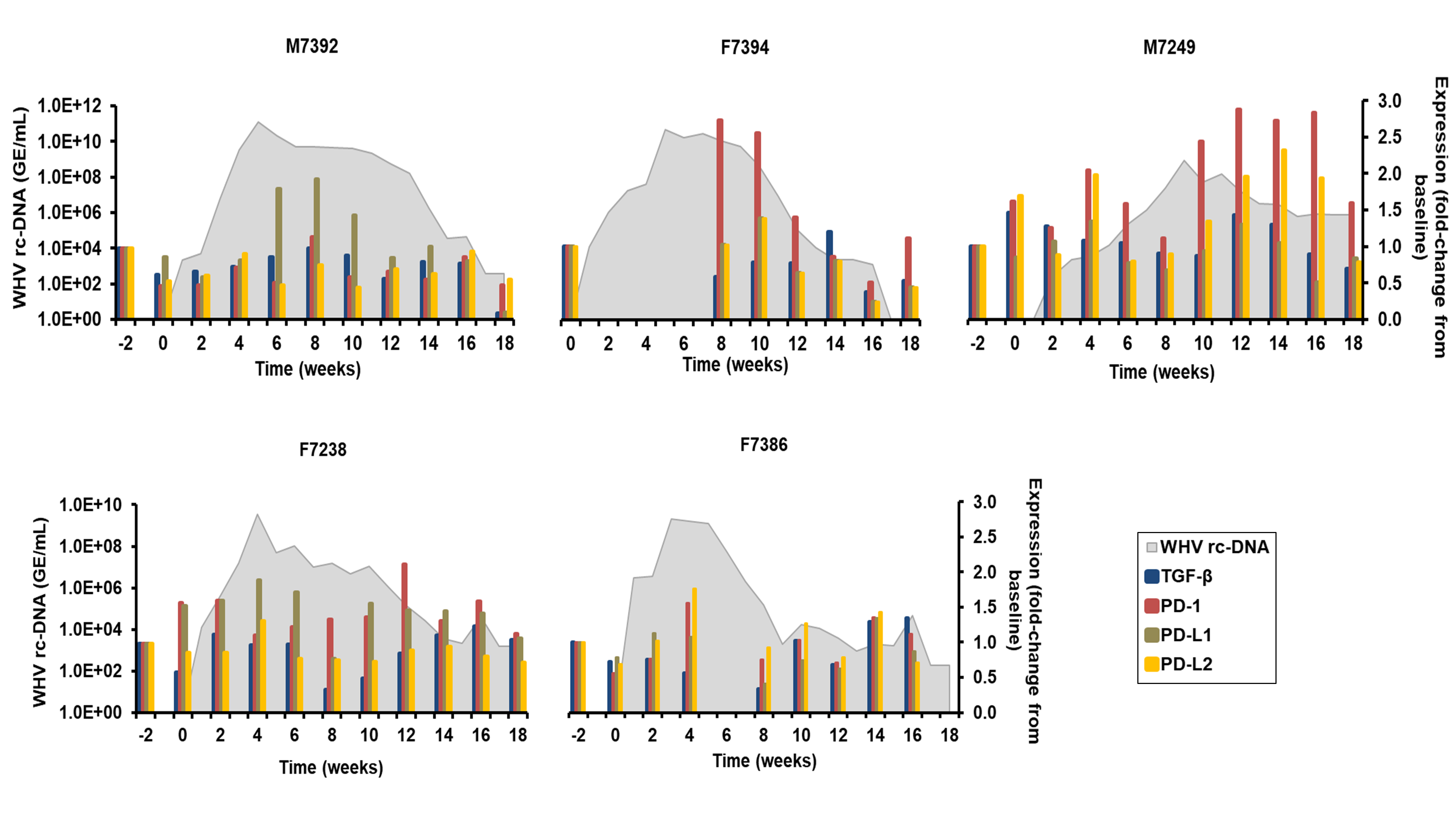

Supplement: S11 Fig — Changes in the expression of TGF-β, PD-1, PD-L1, and PD-L2 in the periphery. The fold-change in transcript level of genes from baseline is plotted on the right y-axis, while serum WHV rc-DNA loads are plotted on the left y-axis. (TIF) [file ppat.1008248.s013.tif]

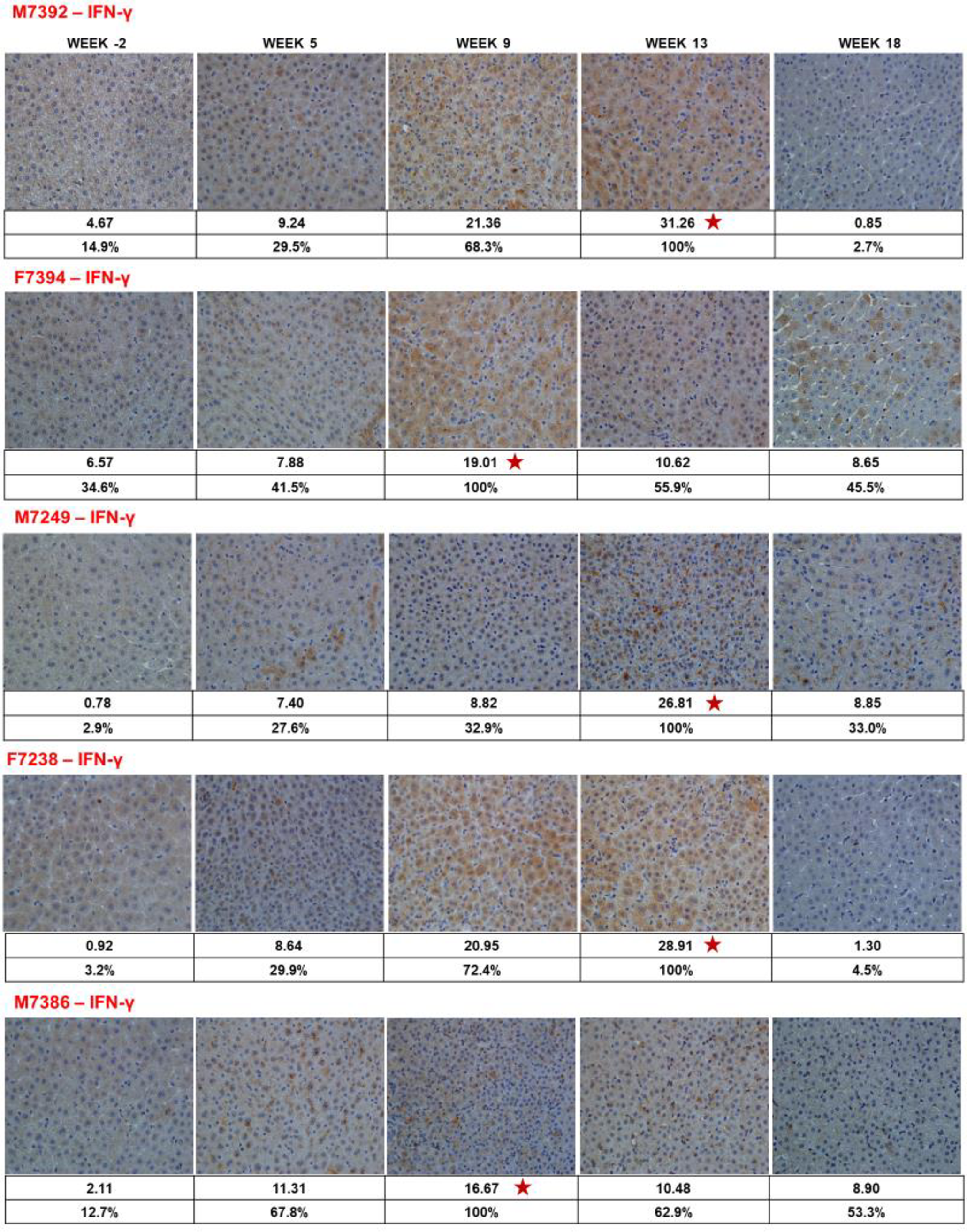

Supplement: S12 Fig — Liver tissues of woodchucks collected at the indicated weeks before and after WHV inoculation were stained with a cross-reactive antibody to IFN-γ. One representative image is shown for each timepoint. The average mean intensity of IFN-γ staining and the relative percentages of staining intensity are provided below each image. The maximum of average mean staining intensity is indicated by an asterisk. (TIF) [file ppat.1008248.s014.tif]

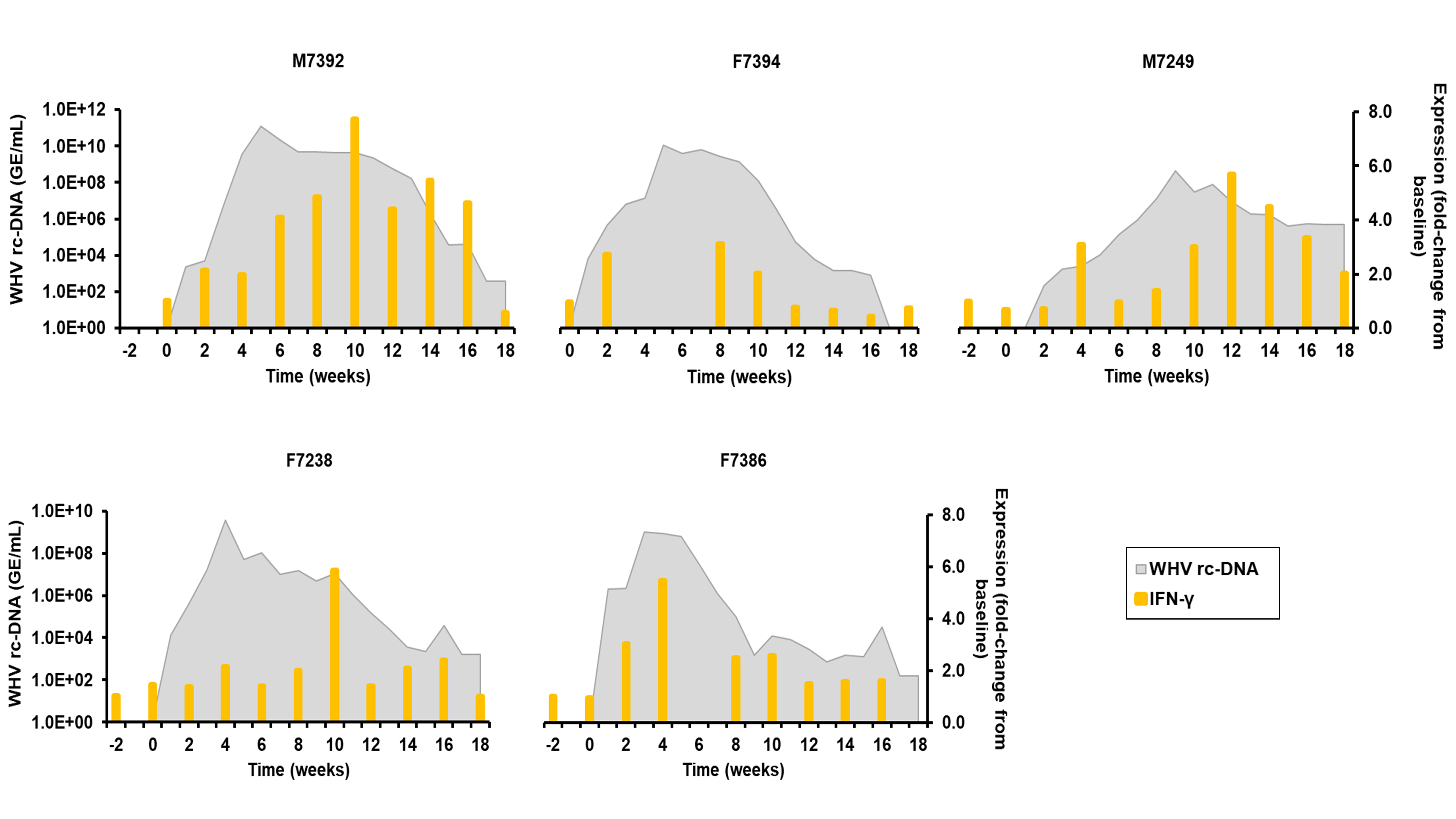

Supplement: S13 Fig — The fold-change in blood transcript level of IFN-γ from baseline is plotted on the right y-axis, while serum WHV rc-DNA loads are plotted on the left y-axis. (TIF) [file ppat.1008248.s015.tif]
